# Mechanistic Insights Into the Thermal Pyrolysis of Branched Polyolefins Using Kinetic Modeling

**DOI:** 10.1002/cssc.202502727

**Published:** 2026-08-30

**Authors:** Aswathy K. Raghu, Alexander K. Best, Varaha Jayarama Krishna Jonnalagedda, Hilal Ezgi Toraman, Paulami Majumdar, Linda J. Broadbelt

**Affiliations:** ^1^ Department of Chemical and Biological Engineering Northwestern University Evanston Illinois USA; ^2^ John and Willie Leone Family Department of Energy and Mineral Engineering Penn State University University Park Pennsylvania USA; ^3^ Department of Chemical Engineering Penn State University University Park Pennsylvania USA; ^4^ Institute of Energy and the Environment Penn State University University Park Pennsylvania USA; ^5^ Materials Discovery and Development Core R&D The Dow Chemical Company Midland Michigan USA

**Keywords:** kinetic modeling, method of moments, polymer pyrolysis, polyolefins

## Abstract

We present a detailed mechanistic analysis of thermal pyrolysis of linear and branched polyolefins. The pyrolytic pathways in high‐density polyethylene (HDPE), polypropylene (PP), and linear low‐density polyethylene (LLDPE) are compared. The mechanistic model for LLDPE is newly developed, based on the population balance approach incorporating reactions on the branches as well as the main chain. Specific reactions are unfurled based on a compact number of reaction families, each of which is governed by a structure/reactivity relationship to specify the activation energy of individual reactions and statistical mechanical estimates to quantify the Arrhenius pre‐factor. The results from the model for LLDPE are compared to new data collected from micropyrolysis experiments that quantify detailed temporal product distributions. PP is confirmed as the most reactive of the three polymers, making the highest amount of low molecular weight products at a given time. For LLDPE, branches serve as positions for hydrogen abstraction, opening up new pathways for deconstructing the main chain. The length of the branches in LLDPE is shown to influence the degradation rate. Understanding the connection between structure and reactivity can help design polymer topologies and pyrolysis reaction conditions to optimize the recycling process and thus the product yields and selectivities.

## 1 Introduction

The generation of plastic waste has been increasing at a considerable rate over the past few decades. Currently, around 400 million tons of plastic waste are generated per year, and at this rate, it is projected to reach 1 billion tonnes by 2050 [1]. The majority of this waste is landfilled, incinerated, or mismanaged, and only about 9% of it is recycled [1]. Bringing about circularity in plastic usage by recycling would enable us to leverage the benefits of plastics while addressing the issue of waste accumulation. At present, mechanical recycling is the most widely used recycling strategy. It is a physical process that recovers and reprocesses plastic waste into new products without significant change to its molecular structure [2]. The downside of mechanical recycling is that contaminants from the waste can get trapped and downgrade the material, especially with multiple recycling loops. This limits the number of times mechanical recycling is feasible for a material. Furthermore, current mechanically recycled products often require a combination of recycled resins with virgin polymers and additives to meet product specifications for high‐performance applications such as packaging materials. Chemical recycling, on the other hand, is the process of chemically transforming the polymer chain into smaller entities and has been gaining attention due to its ability to potentially integrate waste further back in the value chain and generate higher quality products. In some cases, such as glycolysis, chemical recycling methods result in the production of monomers with high selectivity [3, 4]. However, these methods are relevant only for condensation polymers where polymerization can be easily reversed and hence have limited applicability. Plastics such as polyolefins have strong C—C bonds in the backbone that make conversion to individual monomers very difficult [5]. Developing effective methods to recycle them is a necessity as they form a significant constituent of waste plastics owing to their extensive usage in packaging [6, 7]. Pyrolysis is a potential strategy to convert polyolefins to hydrocarbons that can be used as fuels [5, 8]. It is the process of heating the polymer in the absence of oxygen to very high temperatures. This method is reasonably agnostic to the polymer type and can be applied to mixed plastic wastes as well [9].

Pyrolysis of plastics results in a wide range of products, and modeling is an excellent complement to experimental studies to unravel the controlling features of the complex chemistry. Techniques involving various levels of chemistry are employed for modeling pyrolysis [10, 11]. Although global models [12, 13, 14] are the most popular for their simplicity, they are designed to represent the overall conversion to specific lumps of products and do not contain any mechanistic information. Pathways‐level modeling [15, 16, 17, 18] involves a higher level of chemical detail and predicts the product distributions comprised of individual molecule classes or molecules, but it has limited applicability due to the absence of mechanistic features. Mechanistic modeling is the most informative about the reaction pathways and product distributions. Mechanistic modeling methods vary in the level of complexity based on their fundamental approaches. A moments‐based mechanistic model assumes molecular weight distributions for classes of species that are then tracked using their moments. Several works [19, 20, 21, 22, 23, 24, 25] based on this approach have been published, and broadly, the level of accuracy in these models depends on the number of different species classes tracked. The kinetic Monte Carlo method for mechanistic modeling contains the highest level of chemistry by tracking individual chain lengths, but it is computationally expensive [26, 27, 28].

**FIGURE 1:**
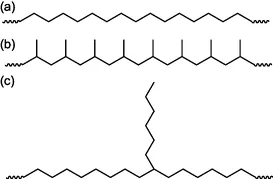
Backbone structure of (a) HDPE, (b) PP, and (c) LLDPE of branch length 6, corresponding to the co‐monomer 1‐octene.

In this work, we present a moment‐based mechanistic model developed for linear low‐density polyethylene (LLDPE) pyrolysis, along with our analysis of its reaction pathways. We compare the reaction pathways in LLDPE with those in high‐density polyethylene (HDPE), which has a linear structure, and polypropylene (PP), which has methyl groups on alternate carbon atoms on the backbone, to draw insights into the pyrolytic pathways in branched polymers. The structures of these polymers are illustrated in Figure 1. HDPE and PP are homopolymers of ethylene and propylene, respectively. Although PP has a high concentration of methyl groups on the backbone, it essentially behaves like a linear polymer because the methyl groups tend to remain on the backbone. On the contrary, the branches on the backbone in LLDPE function as reaction sites and influence its pyrolysis behavior by opening up new reaction pathways. The short‐chain branches in LLDPE are introduced by co‐polymerizing ethylene with alpha‐olefins. The branch length in LLDPE is determined by the type of co‐monomer used for polymerization. The commonly used co‐monomers are 1‐octene (branch length = 6), 1‐hexene (branch length = 4), and 1‐butene (branch length = 2). The branch content in LLDPE is determined by the extent of co‐monomer incorporation into the polymer, and the degree of branching determines the morphology of LLDPE. At low branch content, it is a homogeneous random copolymer that is miscible in the solid phase. At higher branch content, a separate amorphous phase of high branch content forms, and LLDPE becomes a heterogeneous, immiscible, multiphase mixture in the solid state [29]. In order to track the evolution of branches on the backbone and its impact on the reactions, we developed a methodology based on the method of moments to model the reactions when changes also occur on the backbone of the chains, in addition to the ends. In this work, we also present a comparative study of different cases of LLDPE with varying branch content and average branch lengths, using a model that is validated against micropyrolysis experimental data collected as a function of reaction time. Our findings will be helpful in designing the branching in the polymers such that they are more effectively recyclable by pyrolysis.

## 2 Modeling Methodology

### 2.1 Reaction Mechanism

**FIGURE 2:**
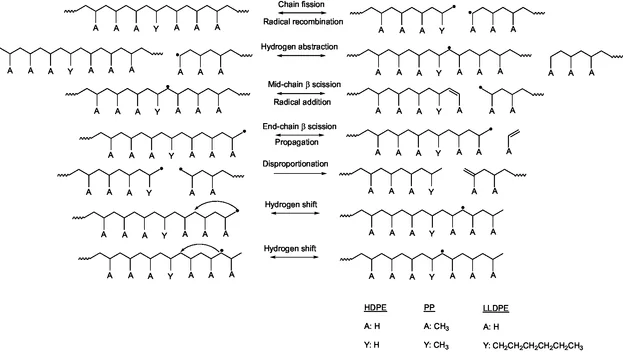
An illustration of the major reaction families that occur during pyrolysis. Chain fission initiates radicals, hydrogen abstraction and mid‐chain beta scission are major contributors to propagation, and recombination and disproportionation cause their termination. The symbol “A” is used to denote the atoms or substituents attached to the C—C backbone, and “Y” is used to indicate that LLDPE has short‐chain branches of the comonomer, with C6 branches used as an example here, with a certain branching density.

Pyrolysis of polyolefins proceeds by a radical mechanism that involves initiation, propagation, and termination steps. The major reaction families involved in the mechanism are illustrated in Figure 2. The reaction families considered are chain fission, radical recombination, hydrogen abstraction (both intermolecular and intramolecular), mid‐chain beta scission, radical addition, end‐chain beta scission, propagation, and disproportionation. In general, the overall reaction pathway is as follows: chain fission initiates the reaction, hydrogen abstraction and mid‐chain beta scission cause propagation of radicals, and radical recombination and disproportionation terminate the radicals. However, additional complexity in the reaction arises due to other kinetically significant reactions, including hydrogen shift reactions that dictate the formation of specific small species. For instance, 1,5 hydrogen shifts result in the formation of a radical on the fifth carbon on the backbone, which can later undergo scission to produce a hexene molecule or a propyl radical.

The pyrolysis of polymers is further complicated by the molecular weight distribution. A polymer sample is composed of chains having varying chain lengths, some chains consisting of thousands of C—C bonds. Breakage of these bonds during pyrolysis results in the formation of thousands of unique species, and their possible reactions are *O*(10^6^). It is computationally prohibitive to track every one of these species separately. In this study, we employ a population balance approach and a solution strategy based on the method of moments.

### 2.2 Method of Moments—Modeling Approach

In this approach, a continuous reaction mixture [30] is considered, where the molecular weight (a function of the chain length) of each species is the variable on which the properties depend. The reactivity of a species is determined by its nature (dead/radical, linear/branched) and the type of its chain ends (saturated/unsaturated). The reaction mixture is divided into species classes according to these features. Each of the species classes comprises chains that have a specific feature in common but span a range of molecular weights. The reactor design equations, in terms of molecular weight distributions of these species classes, form a set of integrodifferential equations. These integrodifferential equations are converted into ordinary differential equations (ODEs) using moment operations [31, 32, 33]. The resultant ODEs have the moments of the distributions as the differential variables. The moments are used to calculate the polymer properties such as number average and weight average molecular weights as a function of time, assuming a batch reactor. Zeroth, first, and second moments are sufficient to extract relevant information about the distributions and are hence tracked in our model [34]. However, two of the reaction families (chain fission and hydrogen abstraction) are chain‐length dependent, and consequently, their lower moments depend on the higher moments. Therefore, the third moments of some species classes are required to bring closure to the system of equations. The Saidel–Katz approximation is used to calculate the third moment as an algebraic variable [22]. In addition to the evolution of overall chain length distributions, our model can also explicitly track the concentrations of certain small species (alkanes and alkenes) up to a specified carbon number. This enables the prediction of small species released from pyrolysis. Detailed derivation of the moment equations is provided in Kruse et al. [22]. Only reactions in the melt phase are considered in the model, that is, once species volatilize, they do not react further, and some higher‐order reactions in the melt phase, such as cyclization and aromatization, are assumed to not occur. This assumption is valid for cases when the volatile products from the reaction are swept away from the reactor and are prevented from reacting further.

### 2.3 LLDPE Model

In the case of HDPE pyrolysis, all the polymer chains undergoing reaction have an identical backbone, and this is also the case for PP, as the methyl groups on the PP backbone stay attached to the backbone as suggested by the product distribution and the relatively slow rate of methyl radical formation. However, that does not hold in the case of LLDPE due to the presence of reactive short‐chain branches on the backbone with the frequency dictated by the branching density. As a result, the same reaction family can result in diverse products depending on the reaction site on the backbone. For instance, chain fission in HDPE always yields two primary radicals, whereas in LLDPE, in addition to the potential formation of two primary radicals, chain fission near a branch point could result in a primary and secondary radical instead. Likewise, hydrogen abstraction can form tertiary as well as secondary radicals, and mid‐ and end‐chain beta scission can result in primary and secondary radicals. Table 1 illustrates the various types of product species formed from the forward reaction families in the three polymers. Modifications are introduced to the moment equations to account for the different backbone structure in LLDPE by introducing a multiplicative factor in the rate expressions that represents the likelihood that a particular reaction will occur. This factor is a function of the average number of branch points and is calculated as described below in Equation (1).

**TABLE 1:** The different types of products formed with an emphasis on those from a branched species considered in the LLDPE model. Products in italics are in the LLDPE model only. Products in plain text are in both the LLDPE and PP (where the “branches” are methyl groups) models, and those in bold are in all three models, HDPE, PP and LLDPE, showcasing the significant complexity of the LLDPE model. Such detail is included only in the forward reactions as they are the dominant reactions.

Reactions of branched species	Different types of species formed as products
Chain fission	2 branched end radicals
	*1 branched end radical, 1 branched mid‐radical*
	1 branched end radical, 1 linear end radical
	*1 branched end radical, 1 linear mid‐radical*
	*1 branched mid‐radical, 1 linear end radical*
	1 branched end radical, 1 small radical
	1 branched mid‐radical, 1 small radical
Mid‐chain beta scission	1 branched end radical, 1 branched dead species
	1 branched mid‐radical, I branched dead species
	1 small radical, 1 branched end radical
	1 small radical, 1 branched mid‐radical
	1 small dead species, 1 branched end radical
	1 small dead species, 1 branched mid‐radical
Hydrogen abstraction	**Primary to primary radical**
	**Primary to secondary radical**
	Primary to tertiary radical
	**Secondary to primary radical**
	**Secondary to secondary radical**
	Secondary to tertiary radical
	Tertiary to primary radical
	Tertiary to secondary radical
	Tertiary to tertiary radical
End‐chain beta scission	1 branched end radical, 1 monomer
	1 branched mid‐radical, 1 monomer
Hydrogen shifts	**Primary to secondary radical and its reverse**
	Primary to tertiary radical and its reverse
	Secondary to tertiary radical and its reverse

Every chain in the branched species classes is assumed to have the same proportion of branch points on its backbone, which is given by the average number of branch points that evolves with time. The change in branches is tracked using the “concentration” of the branches as the differentiable variable. Branch concentration is a quantity that represents the moles of branches per unit volume and is used to calculate the average number of branch points per species. The number of branch points on every branched species, be it dead or radical, is estimated by dividing the total branch concentration by the sum of the zeroth moments of all branched species.

(1) $${\mathrm{Num}}_{\mathrm{Br}\_\mathrm{Pts}}=\;\frac{\text{total branch concentration}}{\text{zeroth moment of all branched species classes}}$$

The number of ends on a branched species is given by

(2) $${\mathrm{Num}}_{\text{Ends}}\;=\;{\mathrm{Num}}_{\mathrm{Br}\_\mathrm{Pts}}\;+\;2$$

Linear species in all three models are separated based on the type of ends because the ends have relevance in some reactions (such as radical addition or specific bond fission). The number of ends that a branched species has depends on the number of branch points, as each branch will have an end in addition to the two backbone ends. Therefore, dividing the species into classes based on types of ends is complex and computationally demanding. Hence, all the branched species are classified based on whether they are a dead or radical species and the position of the radical. Nevertheless, it is important to track the evolution of ends in branched species. Therefore, the end‐chain types on branched species are considered as a property of the branched species and are tracked as a differential variable.

**FIGURE 3:**
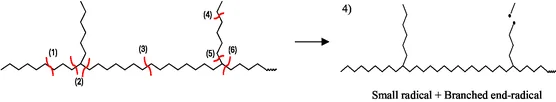
Illustration of different cleavage sites for chain fission on a branched dead species in LLDPE that could lead to different products. Products for case (4) are shown. A detailed diagram showing all the possible product cases is provided in the Supporting Information.

The factor representing the likelihood of a chain fission reaction forming a small radical and a branched end radical (case 4 in Figure 3) is calculated as

(3) $$\begin{array}{c} {\mathrm{Num}}_{\mathrm{Br}\_\mathrm{Pts}}\times \frac{\mathrm{all}\;\mathrm{specific}\;\mathrm{branches}\;\left(\mathrm{sat}/\mathrm{unsat}\;\mathrm{end}\right)\mathrm{with}\;\mathrm{length}\;>=\mathrm{specific}\;\mathrm{length}\;\left(\#C\right)}{\mathrm{total}\;\mathrm{branches}} \end{array}$$

A detailed illustration of the possible reactions and their corresponding algebraic multipliers is presented in the Supporting Information.

In our model, we consider branches to be interspersed with a constant interval and assume a long length of the C—C backbone to be present between the branches, such that whenever a branched species undergoes scission, two long ends are formed. This assumption is valid for LLDPE with low branch content (up to ~ 20 branches/ 1000 carbons). Higher branch contents solicit the need for tracking the end lengths when scission occurs and is beyond the scope of the current population balance model.

Formation of branched species during the reaction is not considered in the model as it is not expected to be significant at typical pyrolysis temperatures. However, in order to account for the termination reactions of mid‐radicals, recombination reactions of the mid‐radicals with end radicals are included in the model, with an approximation that no new branches are formed during those reactions. This is a sensible assumption given the fact that recombination reactions have lower rates compared to radical propagation steps, as they occur between two radical species that are always present in very low concentrations compared to the dead species.

All the small molecules formed from LLDPE are considered to be linear species. An average branch length of 6 is used in the base case of the model, considering the co‐monomer to be 1‐octene.

### 2.4 Tracking the Branches

**FIGURE 4:**
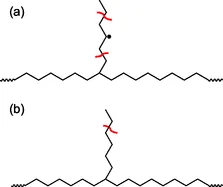
Illustration of two cases of reactions that can cause a branch to break. (a) Mid‐chain beta scission on branch (b) chain fission on branch. Case (a) can result in the formation of a C2 radical or a C5 dead species depending on the direction of scission. The change in branch length will also change accordingly.

Tracking the length and number of branches is crucial in the LLDPE model because it directly influences the depolymerization behavior during pyrolysis. Our model tracks the concentration of branches of different lengths (up to the initial average branch length) as differential variables with the rates of change dictated by the reactions that cause the branches to break. An illustration of two such cases is depicted in Figure 4.

The formation of small species can happen from the ends and also from the branches depending on the length of the branch. In order to track the concentrations of specific branch lengths, whenever a small species whose carbon number is less than the initial branch length is formed, the corresponding rate of reaction is subtracted from the branch concentration of the initial branch length, and the difference is added to the appropriate branch concentrations. This involves the approximation that whenever a branch is cut, it always happens from the initial branch length. This is reasonable because the concentration of initial branch lengths is about an order of magnitude higher than the other branch lengths that form during the reaction. Therefore, if the initial branch length is 6, and a C2 species is formed from a branch, the rate of that reaction is subtracted from the ODE of ${\mathrm{conc}}_{{\mathrm{branch}}_{6}}$, and the same term is added to the ODE of ${\mathrm{conc}}_{{\mathrm{branch}}_{4}}$. This indirectly accounts for the loss of C2 from the branch. Now, the C2 species could be a radical formed by mid‐chain beta scission, and in that case the branch formed has an unsaturated end. Since the concentrations of saturated and unsaturated ends on the branched species are separately tracked, the fractions of unsaturated and saturated ends at a given time are known, and they are used to estimate the approximate change in concentration of specific branches. Below is an example of the calculation for the case where the average branch length is 6, losing a species of length C2.

$$\text{tota}l_{\text{branc}h_{\mathrm{cut}}}=\text{branc}h_{\mathrm{cu}t_{C1}}+\text{branc}h_{\mathrm{cu}t_{C2}}+\text{branc}h_{\mathrm{cu}t_{C3}}+\text{branc}h_{\mathrm{cu}t_{C4}}+\text{branc}h_{\mathrm{cu}t_{C5}}$$

where

$${\mathrm{branch}}_{{\mathrm{cut}}_{C2}}=\;\sum {\mathrm{rate}}_{\mathrm{mid}\;\mathrm{chain}\;\mathrm{beta}\;\mathrm{scissions}\;\mathrm{that}\;\mathrm{make}\;C2\;\mathrm{small}\;\mathrm{radicals}\;\mathrm{from}\;\mathrm{branch}}+\;\sum {\mathrm{rate}}_{\mathrm{mid}/\mathrm{end}\;\mathrm{chain}\;\mathrm{beta}\;\mathrm{scissions}\;\mathrm{that}\;\mathrm{make}\;C2\;\mathrm{dead}\;\mathrm{species}\;\mathrm{from}\;\mathrm{branch}}+\;\sum {\mathrm{rate}}_{\mathrm{chain}\;\mathrm{fissions}\;\mathrm{that}\;\mathrm{make}\;C2\;\mathrm{small}\;\mathrm{radicals}\;\mathrm{from}\;\mathrm{branch}}-\sum {\mathrm{rate}}_{\mathrm{radical}\;\mathrm{recombinations}\;\mathrm{of}\;C2\;\mathrm{small}\;\mathrm{radicals}\;\mathrm{and}\;\mathrm{branch}\;\mathrm{end}\;\mathrm{radical}}$$

$$\frac{d\;{\mathrm{conc}}_{{\mathrm{branch}}_{6_{\mathrm{saturated}\;\mathrm{end}}}}}{dt}=-{\mathrm{total}}_{{\mathrm{branch}}_{\mathrm{cut}}}-\sum {\mathrm{rate}}_{\mathrm{mid}\;\mathrm{chain}\;\mathrm{beta}\;\mathrm{scissions}\;\mathrm{that}\;\mathrm{remove}\;C6\;\mathrm{branch}}-\;\sum {\mathrm{rate}}_{\mathrm{chain}\;\mathrm{fissions}\;\mathrm{that}\;\mathrm{remove}\;C6\;\mathrm{branch}}$$

$$\frac{d\;{\mathrm{conc}}_{{\mathrm{branch}}_{4_{\mathrm{unsaturated}\;\mathrm{end}}}}}{dt}=\;+\;{\mathrm{branch}}_{{\mathrm{cut}}_{C2}}\times \;F_{p_{1}}\;-\sum rate_{\mathrm{mid}\;\mathrm{chain}\;\mathrm{beta}\;\mathrm{scissions}\;\mathrm{that}\;\mathrm{remove}\;C4\;(\mathrm{unsaturated})\;\mathrm{branch}}-\sum {\mathrm{rate}}_{\mathrm{chain}\;\mathrm{fissions}\;\mathrm{that}\;\mathrm{remove}\;C4\;(\mathrm{unsaturated})\;\mathrm{branch}}$$

$$\frac{d\;{\mathrm{conc}}_{{\mathrm{branch}}_{4_{\mathrm{saturated}\;\mathrm{end}}}}}{dt}=\;+\;{\mathrm{branch}}_{{\mathrm{cut}}_{C2}}\times \;F_{p_{1}}\;-\sum {\mathrm{rate}}_{\mathrm{mid}\;\mathrm{chain}\;\mathrm{beta}\;\mathrm{scissions}\;\mathrm{that}\;\mathrm{remove}\;C4\;(\mathrm{saturated})\;\mathrm{branch}}-\sum {\mathrm{rate}}_{\mathrm{chain}\;\mathrm{fissions}\;\mathrm{that}\;\mathrm{remove}\;C4\;(\mathrm{saturated})\;\mathrm{branch}}$$

The following equation is used to calculate the average branch length:

(4) $$\text{Average branch length}=\;\frac{\sum_{i=1}^{\text{max branch length}}\left(\text{Concentration of branch length}\;i\right)\times i}{\sum_{i=1}^{\text{max branch length}}\left(\text{Concentration of branch length}\;i\right)}$$

For chains with a uniform backbone structure, chain fission reactions and mid‐chain beta scission reactions on the backbone occur at random positions, and the partitioning of mass for random scissions is given by $\frac{6}{\left(n\;+\;2\right)\left(n\;+\;3\right)}$, where *n* = 1 (1^st^ moment) [31]. However, such cleavages on a branched species can have both proportional and random characteristics. Partitioning of the mass of the degrading branched species to the products could be random or proportional depending on the location of the reaction. The branches are assumed to be distributed at equal spacing on the main chain. All the cleavages occurring on/near the interior branch points that result in branched species as products are considered to be of random nature, resulting in equal partitioning of mass according to the above equation. On the other hand, cleavages on/near the two branch points at the ends can form a combination of linear and branched species, for which case the mass is partitioned proportionally. The following moment equations are used for the rate of formation of linear and branched species, respectively [22].

(5) $$\int_{0}^{¥}\int_{0}^{fy}x^{n}k_{fb}D_{y}\frac{1}{fy}dx\;dy=k_{fb}\left[\frac{f^{n}}{\left(n+1\right)}\right]D^{n}$$

(6) $$\int_{0}^{\infty }\int_{\left(1-f\right)y}^{y}x^{n}k_{fb}D_{y}\frac{1}{fy}\;dx\;dy=k_{fb}\left[\frac{1}{f\left(n+1\right)}-\frac{{\left(1-f\right)}^{n+1}}{f\left(n+1\right)}\right]D^{n}$$

The factor *f* represents the ratio of the size of linear species to that of the reactant branched species and is approximated in terms of the average number of branch points as

(7) $$f\;=\frac{1}{{\mathrm{Num}}_{{\mathrm{Br}}_{\mathrm{Pts}}}+2}$$

For the case of evolution of small molecules, the mass is partitioned in accordance with the size of the species formed.

### 2.5 PP and HDPE Models

**FIGURE 5:**
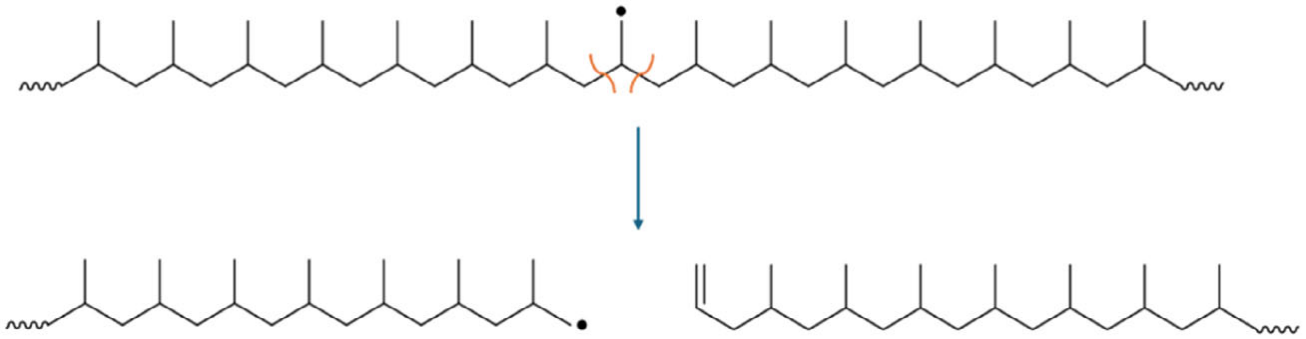
Mid‐chain beta scission from an end radical on the methyl group in the PP model. The formation of a primary radical on the methyl groups and their subsequent scission is an addition to the previously published PP model by Kruse et al. [20].

The HDPE model to which the results of the LLDPE model are compared is adapted from the work of Levine and Broadbelt [24], adding reaction pathways to account for the formation and deconstruction of diolefinic species quantified by new experimental results presented in other work. The PP model used in this work is based on the model developed by Kruse et al. [20]. This model considered abstraction of hydrogen atoms only from the secondary and tertiary positions from the backbone of the chain. Hydrogen abstraction from the methyl groups has a higher activation energy compared to hydrogen abstraction from secondary and tertiary positions, given the same abstracting radical, as it forms a primary radical, and in our earlier work, it was deemed to be kinetically insignificant enough to warrant removal, given that model size was a strong consideration at the time that work was carried out and that excellent prediction of experimental data was achieved [20]. Unlike in the HDPE and LLDPE models, pathways for the formation of diolefinic species are not included in the PP model. This is justified as, based on the experiments conducted in the original study, the yields of diolefinic products were too low to be quantifiable. That said, in the present work, the PP model was modified to allow hydrogen abstraction from the methyl groups on every alternate carbon on the main chain for a fair comparison with LLDPE, in which hydrogen abstraction is allowed from all carbons on the branch. This results in the inclusion of more species and reactions in the model, as the hydrogen abstraction from the methyl groups forms a new class of end‐radical species that can further undergo mid‐chain beta scission to form a new type of unsaturated “head” end that was not considered by Kruse et al. [20]. An illustration of the reaction is shown in Figure 5. Alkanes and alkenes up to C20 are tracked as volatile species in all three models. This assumption was based on both experimental data and consistency across models. Whether a species is considered volatile is based on its boiling point, but because of memory requirements imposed in gPROMS (see below), C20 was the highest carbon number considered for individual products.

### 2.6 Rate Parameters

The rate expressions are based on mass action kinetics for elementary steps. The rate coefficients are of Arrhenius form, which requires pre‐exponential factors and activation energies. These parameters are obtained either from theory (from transition state theory or DFT calculations) or from microscale experiments. In this work, we have used the parameters governing the specification of rate coefficients that were used in the previously published HDPE pyrolysis model [24] as a baseline, tuning some variables to revalidate the HDPE and LLDPE models against new experimental data. As described below and in the Supporting Information, activation energies are calculated using the Evans‐Polanyi relationship, which specifies an intrinsic barrier, *E*_o_, and a transfer coefficient, *α*, for each reaction family, and *E*_a_ values for each reaction that is a member of a given family are specified based on the heat of reaction. The heats of reaction involving tertiary bonds and radicals, which are not present in the HDPE model, were calculated based on the Benson group additivity method. The untuned kinetic parameters used in the model are provided in Table 2, and information about the parameter tuning can be found in the Supporting Information.

**TABLE 2:** Parameters used to calculate the rate coefficients in the models as taken from Levine and Broadbelt [24] as a starting point, and a small set of parameters were optimized against experimental data for both HDPE and LLDPE as described in the Supporting Information. The same values of *A* and *E*_0_ are used for all three polymers to allow for a fair comparison based on structural features alone, where the structural differences are captured by Δ*H*_R_ values that are calculated using Benson group additivity. Of note, the Δ*H*_R_ of fission reactions, which produce a primary and secondary radical, are adapted from DFT calculations conducted by Wu et al. [35].

Reaction type	Frequency factor, *A*, s^−1^ or L mol^−1^ s^−1^	Intrinsic barrier, *E*_0_, kcal mol^−1^	Transfer coefficient (*α*)	Representative Δ*H*_R_, kcal mol^−1^
Chain fission				
Formation of two primary radicals	1.00E+16	2.3	1	87.4
Formation of a primary radical and a secondary radical (i.e., at bonds involving tertiary carbon atoms) in LLDPE on backbone [35]	1.00E+16	2.3	1	73.0
Formation of a primary radical and a secondary radical (i.e., at bonds involving tertiary carbon atoms) in LLDPE to make a low‐molecular weight radical [35]	1.00E+16	2.3	1	70.7
Formation of a primary radical and a secondary radical (i.e., at bonds involving tertiary carbon atoms) in PP on backbone [35]	1.00E+16	2.3	1	74.8
Formation of a primary radical and an allylic radical (Allyl chain fission)	1.00E+16	2.3	1	74.8
Radical recombination	1.10E+11	2.3	0	NA
Disproportionation	1.10E+10	2.3	0	NA
End‐chain beta scission				
HDPE	1.29E+13	11.4	0.76	22.3
PP	1.29E+13	11.4	0.76	21.9
Mid‐chain beta scission				
Secondary to secondary	8.00E+14	11.4	0.76	21.8
Secondary to primary	8.00E+14	11.4	0.76	22.8
Tertiary to primary	8.00E+14	11.4	0.76	23.4
Radical addition	2.88E+07	11.4	0.24	−22.8
Hydrogen abstraction				
Primary to secondary	5.00E+08	12	0.5	−2.8
Primary to tertiary	5.00E+08	12	0.5	−5.9
Secondary to tertiary	5.00E+08	12	0.5	−3.1
Primary to allylic secondary	5.00E+08	12	0.5	−12.5 (tuned)
Secondary to allylic secondary	5.00E+08	12	0.5	−9.5 (tuned)
Intramolecular hydrogen shift of end‐chain radicals				
1,4 hydrogen shift	1.58E+11	22.2	0.5	−2.8 or −5.9 or −3.1
1,5 hydrogen shift	1.82E+10	16.1	0.5	−2.8 or −5.9 or −3.1
1,6 hydrogen shift	1.05E+10	19.7	0.5	−2.8 or −5.9 or −3.1
1,7 hydrogen shift	3.00E+09	20.7	0.5	−2.8 or −5.9 or −3.1
Intramolecular hydrogen shift of mid‐chain radicals				
*x*, *x* + 3 hydrogen shift	1.00E+11	22.2	0.5	0 or −3.1
*x*, *x* + 4 hydrogen shift	1.26E+10	16.1	0.5	0 or −3.1
*x*, *x* + 5 hydrogen shift	7.24E+09	19.7	0.5	0 or −3.1

The rates of reaction are also affected by structural characteristics of the reactants beyond those characterized by different heats of reaction. Algebraic parameters multiply the rate equation to incorporate chain length dependency of termination reactions due to diffusion limitations, which is implemented by the simplified form of Smoluchowski's equation as in Kruse et al. [22].

(8) $$k_{t}=k_{t}^{0}\left(\frac{1}{i^{\gamma }}+\frac{1}{j^{\gamma }}\right)/2$$

where *i* is the size of the reacting species.

Hydrogen abstraction is also known to depend on the size of the abstracting radical and is incorporated in the model as

(9) $$k_{\mathrm{tr}}=k_{\mathrm{tr}}^{0}\left(\frac{1}{i}\right)$$

The parameter values used to run the LLDPE model are listed in Table 3, and the performance of the LLDPE model is assessed against isothermal micropyrolyzer data at two different temperatures, 673.15 and 703.15 K. The comparative study of the three polymers was done for reaction at isothermal conditions at 673.15 K, as it is a reasonable temperature at which the reaction can be tracked using long enough time horizons for all three polymers. The comparative trends reported here are expected to be similar at higher temperatures too, although the degradation would occur at a faster rate.

**TABLE 3:** Inputs required for the LLDPE model. Values in bold are the values used in the base case model.

Initial conditions required in the model	Values used in the current study
*M_n_*—Number average molecular weight	62,500 g/mol
*M_w_*—Weight average molecular weight	125,000 g/mol
Polymer mass density	948.5 kg m^−3^
Average branch length	**6**, 4, 2
Branch content	**10 branches/1000 C**, 20 branches/1000 C
Temperature	Isothermal at **673.15** and 703.15 K

## 3 Experimental Methods

### 3.1 Chemicals and Standards

LLDPE with a nominal granular size of 3 mm was supplied by The Dow Chemical Company (U.S.). The polymer sample was milled to a powder form in a 6775 Freezer/Mill cryogenic grinder (SPEX SamplePrep, Metuchen, U.S.). The particle sizes between 53 and 125 were obtained by sieving LLDPE in powder form. Analytical gases (helium, nitrogen, and air) were purchased at a minimum purity of 99.99% (Praxair, U.S.). Liquid nitrogen was used in GC × GC cryotrap (Praxair, U.S.). Benzene, toluene, and n‐decane with a minimum purity of 99.99% (Sigma Aldrich, U.S.) were used in the quantification of the pyrolyzates using GC × GC–FID.

### 3.2 Micropyrolyzer Coupled to Two‐Dimensional Gas Chromatography

Fast pyrolysis experiments were performed in an Rx‐3050TR Tandem microreactor (Frontier Lab, Japan) coupled to a GC × GC for the online analysis of the pyrolysis products. A polymer sample size of 150 µg was weighed in an XPR26 microbalance (Mettler Toledo, U.S.) and inserted in an 80 µL cup (Frontier Lab, Japan). The sample cup was dropped into the microreactor at pyrolysis temperatures of 673.15 and 703.15 K. The reactor interface was maintained at 300 °C to avoid condensation of pyrolysis products. The flow rate of helium was fixed at 100 mL min^−1^. The pyrolysis vapors were passed through MJT‐1035E Microjet Cryo‐trap (Frontier Lab, Japan) and held for 3 min to improve the separation and peak shape of low‐boiling‐point compounds.

The composition of pyrolysis vapors was analyzed using a GC × GC (Agilent 7890A GC, Santa Clara, U.S.) with an Insight flow modulator (Sepsolve, Peterborough, UK) interfaced with FID (Agilent, Santa Clara, U.S.) and a BenchTOF‐Select mass spectrometer (Markes, Llantrisant, UK). A normal column combination was used to separate the pyrolysis products. The first dimension (Rtx DHA) and second dimension (Rxi‐17 Sil) columns were purchased from Restek (Bellefonte, USA) with dimensions of 60 m × 0.20 mm × 0.5 µm and 8 m × 0.32 mm × 0.25 µm, respectively. The various parts of the flow modulator include the first‐ and second‐dimension columns, a sample loop, a controlled supply of helium from auxiliary lines using a Pneumatic Control Module (PCM), and capillary bleed and transfer lines. The FID temperature was maintained at 300 °C, and the data acquisition was set to 200 Hz. Both the TOF‐MS transfer line and ion source were maintained at 250 °C, the data acquisition was set to 60 Hz, and the mass range (m/z) scan was set between 35 and 600. The pyrolysis products were identified and integrated using ChromSpace software (Sepsolve, Peterborough, U.K.) and NIST 11 MS search version 2.0g. The weight of a compound, wt*_i_*, can be defined as follows:

(10) $${\mathrm{wt}}_{i}=\frac{\mathrm{RF}\cdot \mathrm{FID}\;{\mathrm{area}}_{i}\cdot {\mathrm{wt}}_{\mathrm{std}}}{\mathrm{FID}\;{\mathrm{area}}_{\mathrm{std}}}$$

where wt*_i_* and wt_std_ are the weight of compound *i* and the standard, respectively; FID area*_i_* and FID area_std_ are the flame ionization detector areas of compound *i* and the standard, respectively; and RF is the response factor, described in Equation (12) below.

The calibration curve of the standard corresponds to

(11) $$m=\frac{\mathrm{FID}\;{\mathrm{area}}_{\mathrm{std}}}{{\mathrm{wt}}_{\mathrm{std}}}$$

where *m* is the slope of the calibration conducted with the standard.

From Equations (9) and (10), the wt*_i_* is

(12) $${\mathrm{wt}}_{i}=\frac{\mathrm{RF}\cdot \mathrm{FID}\;{\mathrm{area}}_{i}}{m}$$

The RF can be defined as

(13) $$\mathrm{RF}=\frac{{\mathrm{MW}}_{i}\cdot {\mathrm{ECN}}_{\mathrm{std}}}{{\mathrm{MW}}_{\mathrm{std}}\cdot {\mathrm{ECN}}_{i}}$$

where MW*_i_* and MW_std_ are the molecular weight of compound *i* and the standard, respectively, and ECN*_i_* and ECN_std_ are the effective carbon number for compound *i* and the standard, respectively.

Therefore, the weight of compound *i* can be defined as follows:

(14) $${\mathrm{wt}}_{i}=\frac{{\mathrm{Mw}}_{i}\cdot {\mathrm{ECN}}_{\mathrm{std}}}{{\mathrm{Mw}}_{\mathrm{std}}\cdot {\mathrm{ECN}}_{i}}\cdot \frac{\mathrm{FID}\;{\mathrm{area}}_{i}}{m}$$

The yield wt.% is calculated as follows:

(15) $$\mathrm{wt}.{\%}_{i}=\frac{{\mathrm{wt}}_{i}}{{\mathrm{wt}}_{\mathrm{sample}\;\mathrm{pyrolyzed}}}\cdot 100$$

where wt.%*_i_* is the yield of compound *i*, and wt_sample pyrolyzed_ is the initial mass of polymer sample.

## 4 Results and Discussion

### 4.1 Comparison of LLDPE Model With Experimental Results

The validity of the mechanistic model for LLDPE was tested by comparing the simulation outputs with experimental pyrolysis data from a micropyrolyzer coupled to two‐dimensional chromatography, along with flame ionization and time‐of‐flight spectrometer detectors (Py–GC×GC–FID/TOF–MS). The simulated sample *M*_*n*_ and *M* *_w_* were assumed to be the base case values (Table 3), and the density of the polymer sample was assumed to be 948.5 kg m^−3^. The specific details about the structure of the LLDPE sample used in the experiments were not analyzed, so a branch length of 6 and a branch content of 10 branches/1000 carbons were used in the simulation to represent a commonly available LLDPE. Due to memory constraints of the gPROMS solver, low molecular weight alkanes, alkenes, and dienes up to carbon numbers 16, 19, and 19, respectively, are tracked in this model explicitly.

**FIGURE 6:**
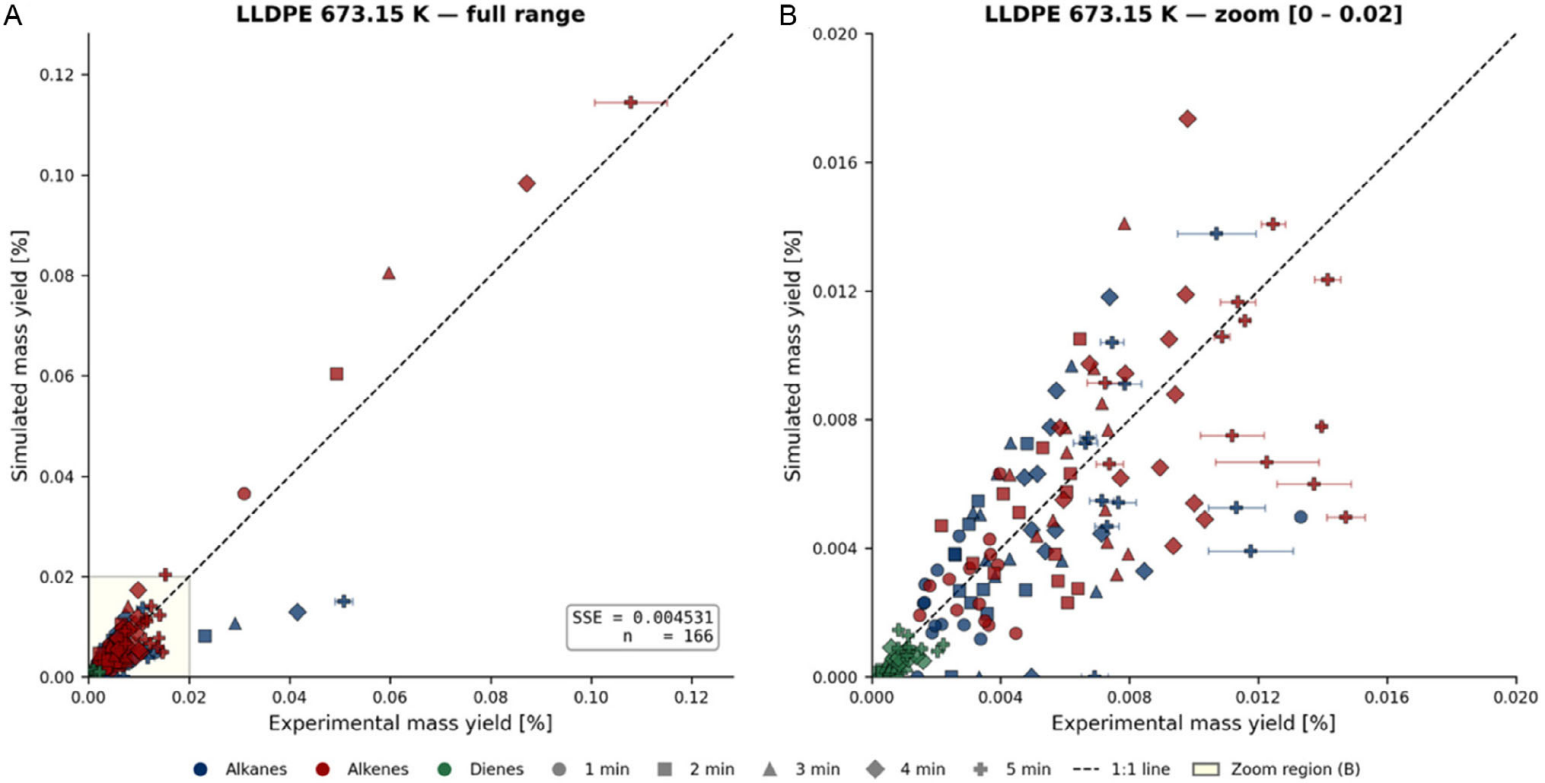
Parity plot comparing the simulation results to experimental results after 1, 2, 3, 4, and 5 min of pyrolysis time at 673.15 K. The color of the data point differentiates the product class: alkane (blue), alkene (red), diene (green). The shape of the data point indicates the pyrolysis time 1 min (circle), 2 min (square), 3 min (triangle), 4 min (diamond), 5 min (cross). Plot A is the entire range of product yields, while plot B zooms in on the 0 to 0.02 wt% product mass yield range.

**FIGURE 7:**
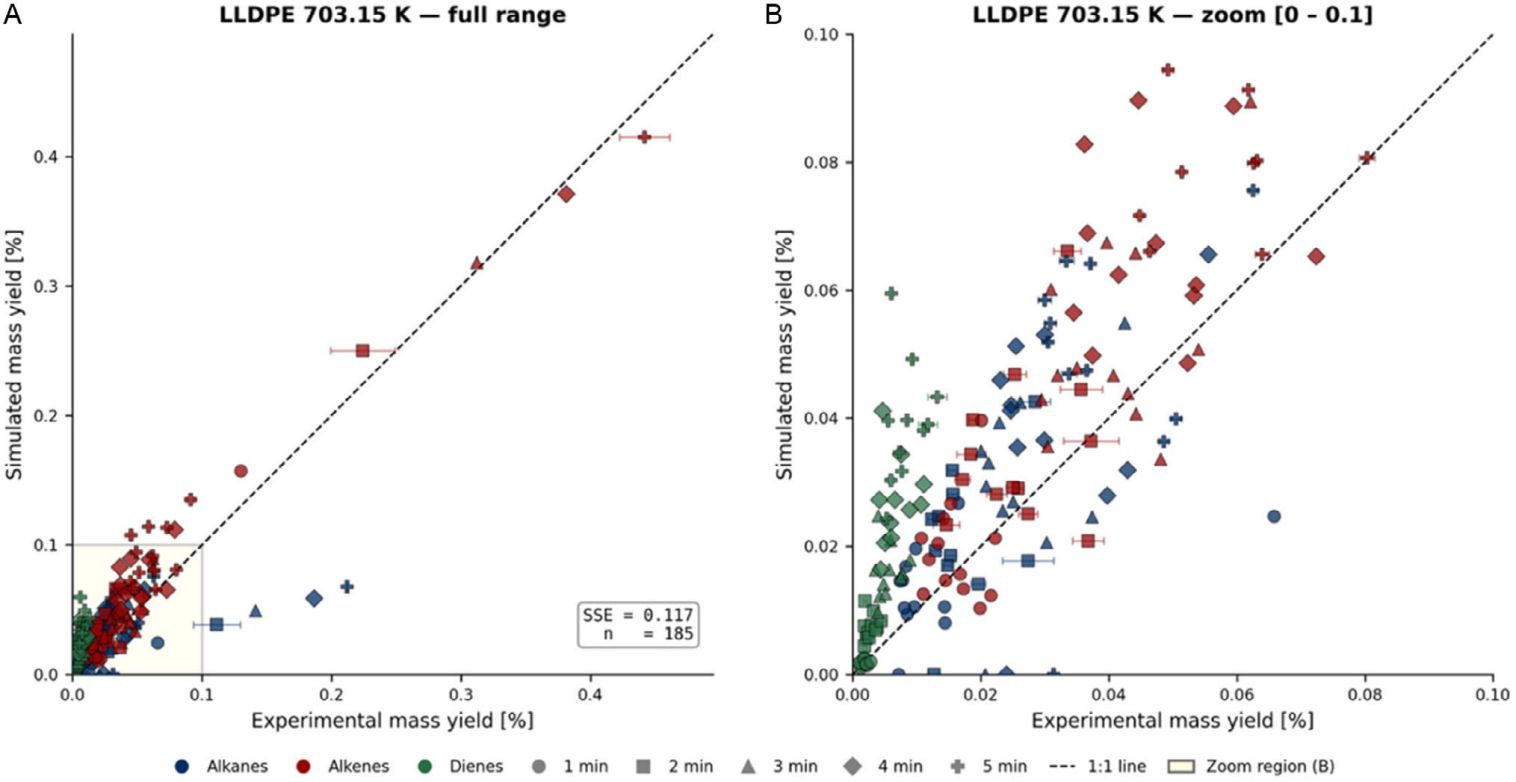
Parity plot comparing the simulation results to experimental results after 1, 2, 3, 4, and 5 min of pyrolysis time at 703.15 K. The color of the data point differentiates the product class: alkane (blue), alkene (red), diene (green). The shape of the data point indicates the pyrolysis time 1 min (circle), 2 min (square), 3 min (triangle), 4 min (diamond), 5 min (cross). Plot A is the entire range of product yields, while plot B zooms in on the 0 to 0.1 wt% product mass yield range.

Figures 6 and 7 depict parity plots comparing the experimental mass yields (*x*‐axes) to the simulated mass yields (*y*‐axes) of the various products collected after 1, 2, 3, 4, and 5 min of pyrolysis time at 673.15 and 703.15 K, respectively. Pyrolysis time is defined based on the following procedure: the sample cup was dropped into the microreactor at pyrolysis temperatures of 673.15 and 703.15 K; the evolved products were collected for 1, 2, 3, 4, or 5 min to capture the temporal evolution of the partial pyrolysis products. The reactor was cooled, and the carrier gas flow was diverted to the bottom of the reactor to prevent further reaction. Under these timescales, LLDPE does not undergo significant deconstruction such that the quantified product yields never exceed 0.5 weight percent (wt%). At both temperatures, the model outputs are closely aligned with the experimental results for all the product classes, alkanes (blue), alkenes (red), and dienes (green), as a function of time. In Figure 6, at 673.15 K, the sum of squared error for the 166 quantified data points is 0.004, with hexane having the largest absolute deviation of 0.036 wt%. Similarly, in Figure 7, at 703.15 K, the sum of squared error for the 185 quantified data points is 0.117. Again, the largest absolute deviation is for hexane, with the model underpredicting the mass yield by 0.14 wt%. A continuation of the comparison between the LLDPE model outputs and the experimental results can be found in the Supporting Information. Specifically, Figures S1 and S2 present a series of bar charts comparing the low‐molecular weight product yields predicted by the model to the experimental measurements as a function of time and temperature. Overall, the model is in good agreement with the experimental trends and product yields at both temperatures and across all time points.

### 4.2 Comparison of LLDPE With HDPE and PP

**FIGURE 8:**
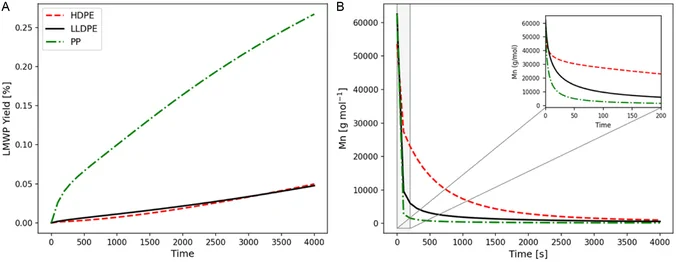
(A) Low‐molecular‐weight product (LMWP) yield and (B) number average molecular weight (*M*_*n*_) decay trend as a function of time for LLDPE, HDPE, and PP. PP has faster evolution of LMWPs than HDPE and LLDPE, while HDPE has the slowest *M*_*n*_ decay.

With the new LLDPE model validated against temporal experimental data, we next compared the degradation characteristics of LLDPE with PP and HDPE. The results of the HDPE model were validated against experimental data as summarized by Best [36], and the comparison of the model for PP to experimental results was previously carried out by Kruse et al. [20]. To demonstrate the effect that the backbone structure has on reactivity, we compare the reaction pathways during isothermal pyrolysis of three structurally different polymers—HDPE, LLDPE, and PP. Figure 8A,B depict the low molecular weight product (LMWP) evolution and *M*_*n*_ decay trend, respectively, as a function of time for the three polymers, HDPE, LLDPE, and PP, undergoing pyrolysis at 673.15 K. The trends observed are indicative of differences in the reaction pathways. The LMWP evolution is highest from PP pyrolysis, followed by LLDPE and HDPE. While LLDPE initially generates more LMWPs than HDPE, once the branches have fully decomposed, HDPE oligomer yields surpass that of LLDPE. This is consistent with the trends reported in the literature [37], but our model specifically shows the mechanism for this observation.

**FIGURE 9:**
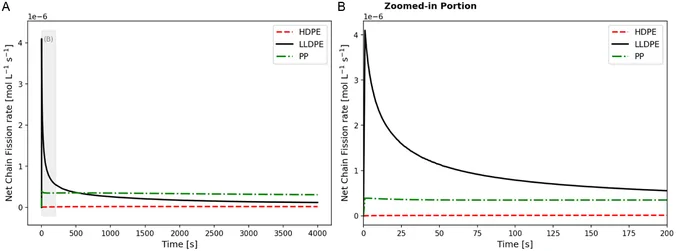
A comparison of the net rate of chain fission in the HDPE (dashed red), PP (dash/dot green), and LLDPE (solid black) systems. On the right, the first 200 s of simulated pyrolysis are depicted.

The *M*_*n*_ decay trend, shown in Figure 8B, demonstrates that PP decays the fastest, followed by LLDPE and HDPE. This is due to the methyl groups on every other carbon on the backbone of a PP chain and the branch points of LLDPE, being a tertiary carbon. The bonds involving tertiary carbon atoms have lower bond strength and are more facile to undergo chain fission that initiates the radical chemistry in pyrolysis. Thus, radical initiation occurs faster in PP and LLDPE than in HDPE, which only has secondary carbons in the backbone. The end radicals formed from fission abstract hydrogen from the backbone or branches of dead chains to make mid‐radicals that decay by mid‐chain beta scission reactions. Although the experimental work reported in this work focused on LLDPE at low conversion using micropyrolysis in order to validate the model, the results in Figure 8B are a testable prediction if samples corresponding to the selected initial conditions were reacted for long reaction times and high‐temperature GPC were available. The comparison of the net rate of fission between the three polymers is depicted in Figure 9. As predicted by DFT calculations [35], the activation barrier for fission at branch points is lower in LLDPE than PP, leading to a higher rate of fission in LLDPE until the average number of branch points decreases to three branches per chain after ~500 s.

**FIGURE 10:**
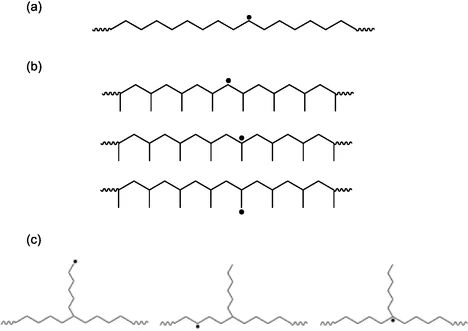
Illustration of mid‐chain radicals formed on the backbone of (a) HDPE and LLDPE (on regions between branches), (b) PP, and (c) LLDPE. Only secondary radicals form in HDPE. All primary, secondary, and tertiary radicals can form in both PP and LLDPE.

The end‐chain radicals that are produced via chain fission can subsequently abstract a hydrogen atom, leading to various types of mid‐radicals. In the case of HDPE, only secondary radicals are formed on the backbone, whereas in LLDPE, there are occasional tertiary radicals in addition to the secondary radicals. These tertiary radicals in LLDPE can form either by bimolecular hydrogen abstraction or by hydrogen shifts from the branches. In the case of both LLDPE and PP, primary, secondary, and tertiary radicals are produced (Figure 10).

**FIGURE 11:**
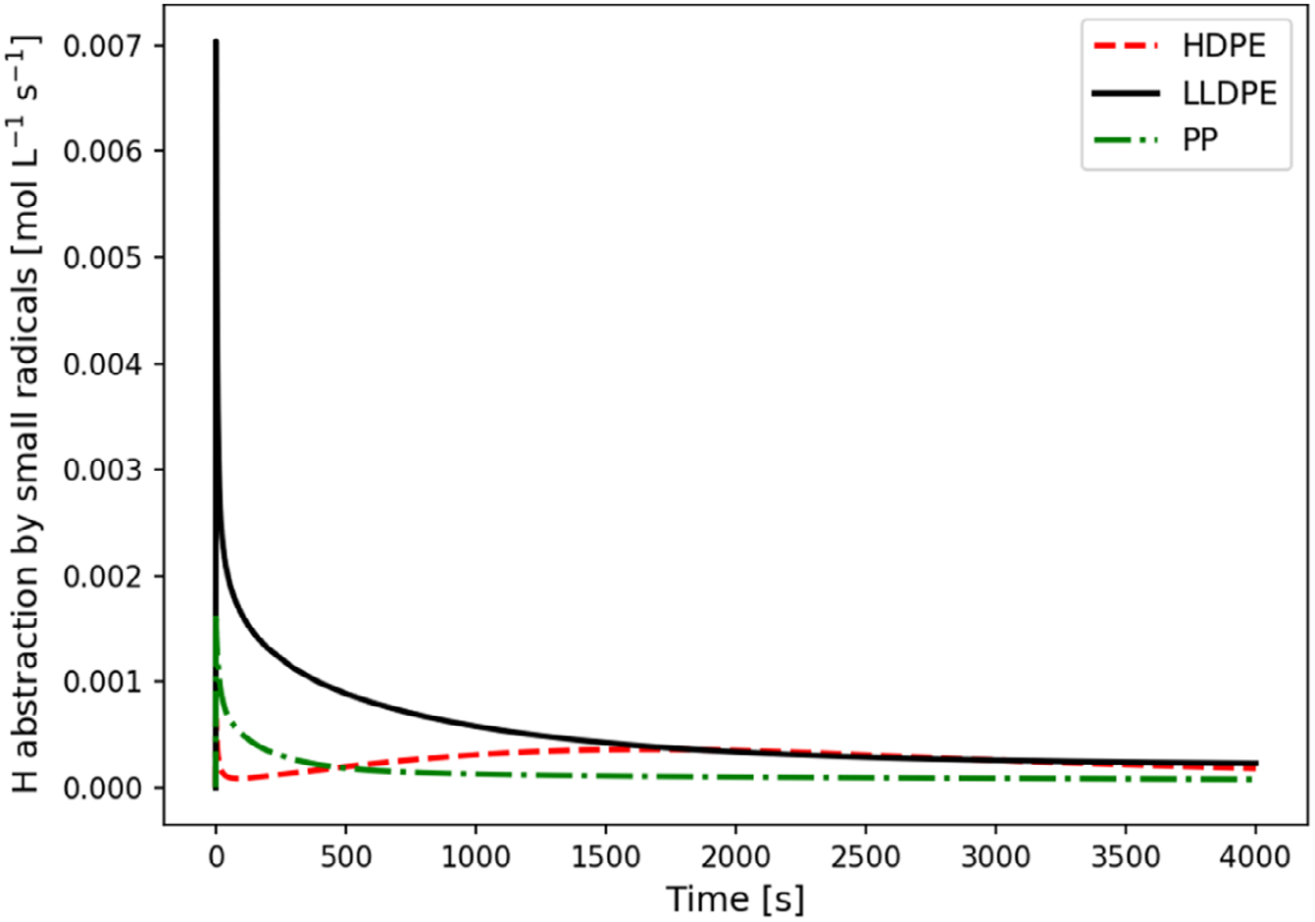
Comparison of net rates of hydrogen abstraction by low‐molecular weight radical species in HDPE (dashed red), PP (dashed/dot green), and LLDPE (solid black).

The net rate of hydrogen abstraction by low‐molecular weight radicals differs between the three systems due to the differences in the rates of chain fission and associated radical concentrations. While the net rate in LLDPE initially spikes as low‐molecular weight radicals are produced from chain fission at branch points, it decreases as the concentration of remaining branches is reduced. Alternatively, the net rate of hydrogen abstraction in PP decreases due to the rapid reduction in *M*_*n*_, which is associated with a lower concentration of abstractable hydrogens as the average chain length decreases. Lastly, the rate of hydrogen abstraction in HDPE increases slowly and to a lesser extent because the rate of formation of radicals is also slower (Figure 11).

**FIGURE 12:**
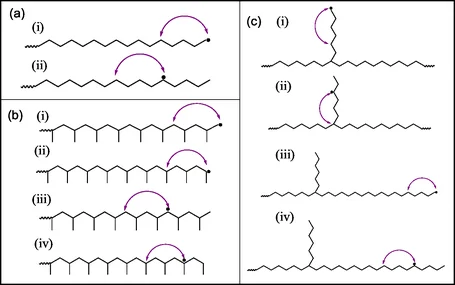
Illustration of some (*x*, *x* + 4) hydrogen shift reactions that are possible in (a) HDPE, (b) PP, and (c) LLDPE. The transformation of radicals in this type of shift is shown. The reactions in HDPE (a) are also possible on the linear portions of the chains in LLDPE.

**FIGURE 13:**
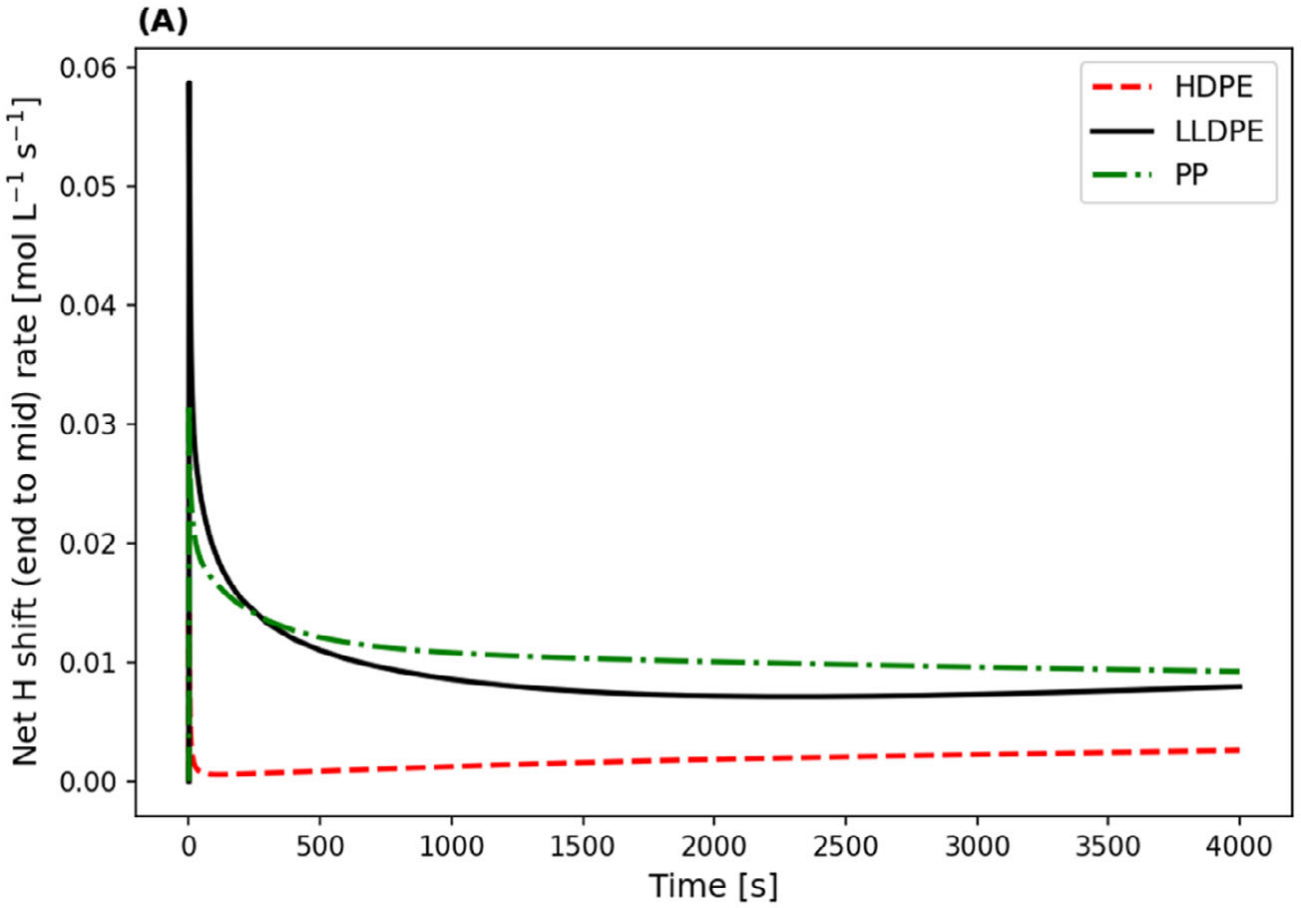
Net rate of hydrogen shifts from end to mid radicals in the three polymers. The hydrogen shift reactions are considered to occur to the fourth, fifth, sixth, and seventh carbon atoms from the end. The possible transformations of radicals are as follows: HDPE—primary to secondary, PP—primary to secondary/tertiary and secondary to secondary/tertiary, LLDPE—primary to secondary and primary to tertiary on a branch (with a probability).

The pathway to produce low‐molecular‐weight products that escape to the volatile phase is by mid‐chain beta scission of radicals formed near the ends of the polymer chains or branches. Apart from bimolecular hydrogen abstraction, such specific radicals are also formed by hydrogen shift reactions from end radicals. These intramolecular hydrogen shifts are favored to occur on certain positions close to the end radical that are determined by steric factors and the stability of the transition state involved in the reaction. Several possible (*x*, *x* + 4) hydrogen shifts that can occur in the three polymers are illustrated in Figure 12. Subsequent beta scission reactions from these specific radical positions do not cause a significant reduction in *M*_*n*_, as small molecules that cleave off from the ends of the polymer chains have a considerably shorter chain length. Hydrogen shift followed by scission (called backbiting) is an important pathway to form small molecules in pyrolysis, as was reported previously by Levine and Broadbelt [24]. The hydrogen shift rates shown in Figure 13 initially increase as the concentration of end radicals increases and later slow down as the size of the species decreases [24]. Hydrogen shift rates in PP are consistently higher than those in HDPE (Figure 13) due in part to the tertiary positions on the backbone. This is one of the reasons why the low molecular weight product evolution initially accelerates in PP.

### 4.3 Comparison of LMWPs

**FIGURE 14:**
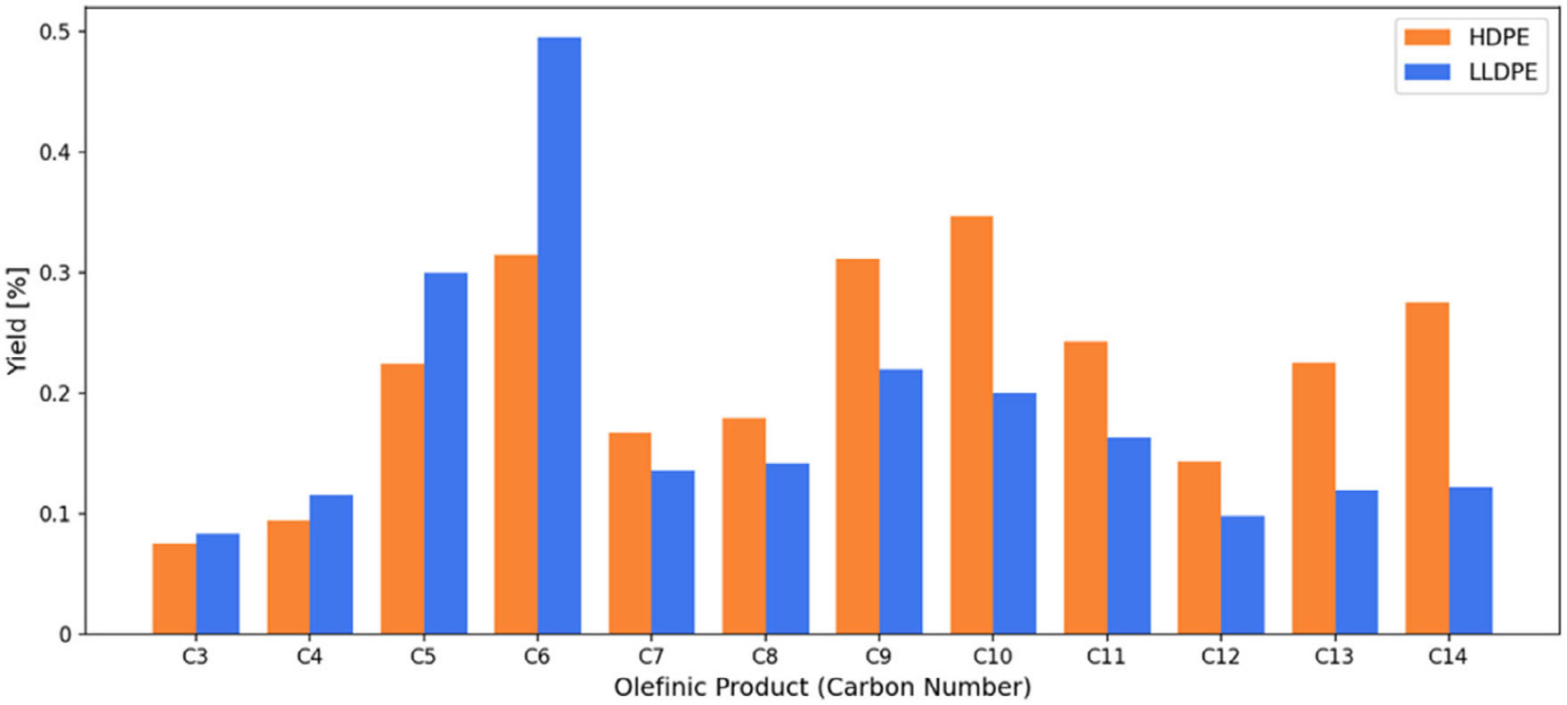
Product distributions (mass yield) of alkenes from LLDPE and HDPE at 4000 s for isothermal pyrolysis at 673.15 K. The mass yields of propene, butene, pentene, and 1‐hexene are higher in LLDPE, whereas the rest of the alkenes have a higher yield in HDPE based on the products in this carbon number range.

**FIGURE 15:**
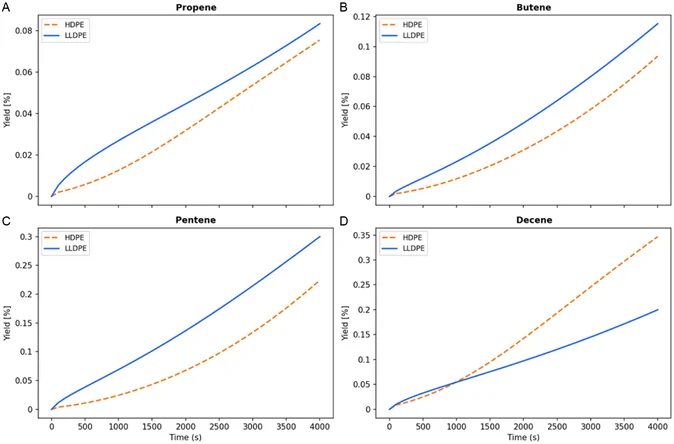
Evolution of mass yields of different species in LLDPE and HDPE. (A) propene, (B) butene, (C) pentene, (D) decene.

Since the products formed from HDPE and LLDPE are similar in structure, we compare the evolution of LMWP from these polymers in this section. Figure 14 shows the predicted alkene product distributions from LLDPE and HDPE at 4000 s, after complete conversion has occurred, at 673.15 K, and Figure 15 shows the temporal evolution of four of the alkene products in detail. At these conditions, all but four alkene yields are observed to be higher during HDPE pyrolysis than during LLDPE pyrolysis. The four exceptions, propene, butene, pentene, and 1‐hexene, are a direct result of branch decomposition. Through the dominant *x*,*x* + 4 hydrogen shift, most branch‐end radicals shift to the C5 position. This will either yield 1‐hexene via beta‐scission, or they can undergo a subsequent *x*,*x* + 3 hydrogen shift to the C2 position. In turn, the C2 radical will decompose to propene. Additionally, hydrogen abstraction from mid‐branch carbons can lead to C3 and C4 radicals, which in turn produce butene and pentene from beta‐scission, respectively. The observations so far suggest that the branch characteristics influence the pyrolysis mechanism of LLDPE. In the next section, we examine these effects in detail.

### 4.4 Effect of Branch Characteristics

#### 4.4.1 Effect of Branch Content

**FIGURE 16:**
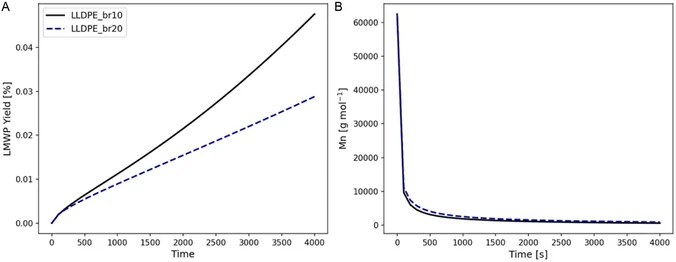
Comparison of two branch contents (20 branches per 1000 carbons and 10 branches per 1000 carbons) in LLDPE with average branch length = 6. (A) LMWP yield and (B) *M*_*n*_ decay trend.

The effect of branch content in the pyrolysis behavior of the LLDPE was examined by comparing two cases—branch contents of 10 and 20 branches/1000 carbons with an average branch length = 6. Figure 16 shows the *M*_*n*_ decay and LMWP yield predicted for these cases. Increasing the branch content is found to slightly slow down the reaction and thus lead to lower LMWP yield. This is an anticipated result as the formation of longer radicals from scission and tertiary radicals by hydrogen shift reactions outweighs the effect of a larger proportion of tertiary carbon atoms where lower‐barrier chain‐fission can occur.

#### 4.4.2 Effect of Branch Length

**FIGURE 17:**
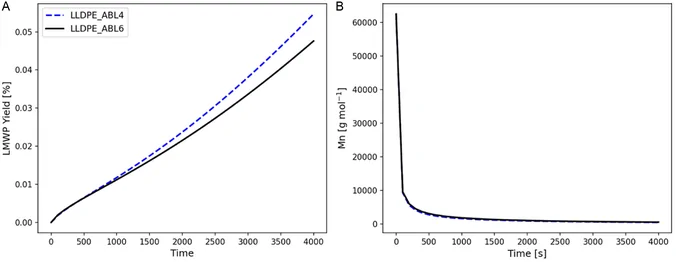
Comparison of different initial average branch lengths of 4 (blue/dashed) and 6 (black/solid), all at 10 branches/1000 carbons for LLDPE pyrolysis. (A) LMWP yield and (B) *M*_*n*_ decay trend.

**FIGURE 18:**
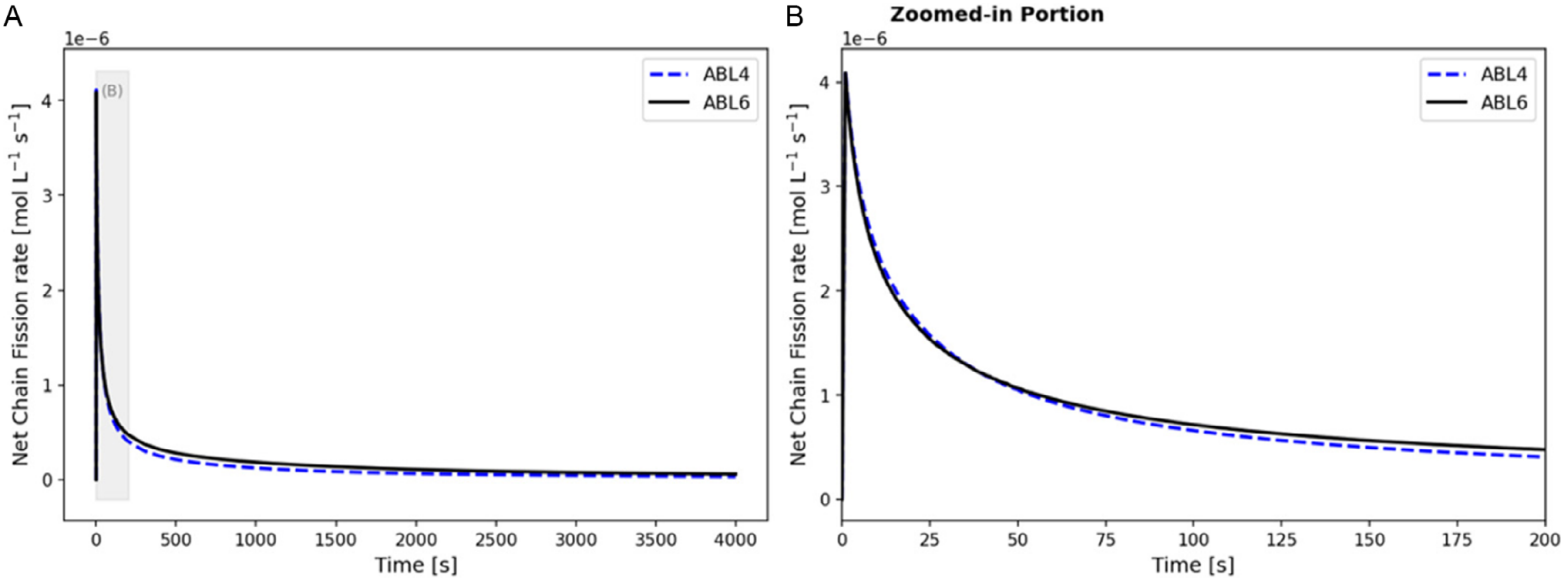
Comparison of the net rate of chain‐fission at branch points for two average branch lengths, ABL4 (blue/dashed) and ABL6 (black/solid).

**FIGURE 19:**
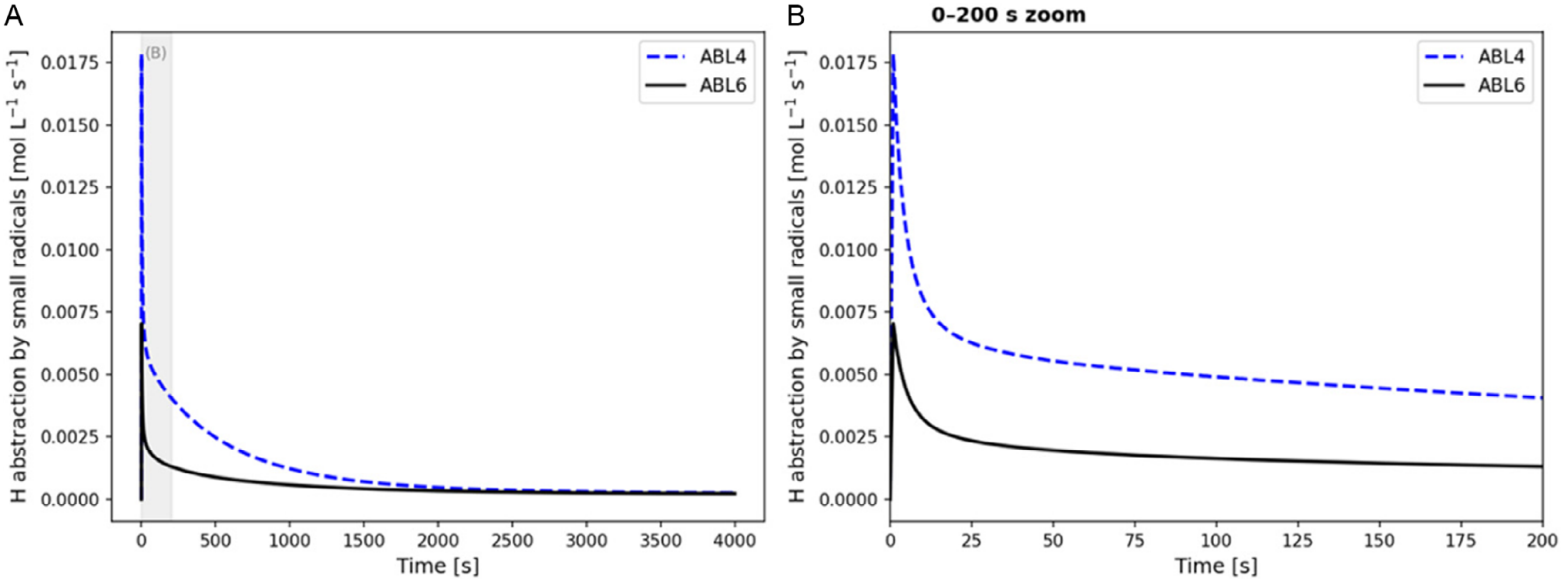
Comparison of the net rate of hydrogen abstraction by a low‐molecular weight radical for two average branch lengths, ABL4 (blue/dashed) and ABL6 (black/solid).

**FIGURE 20:**
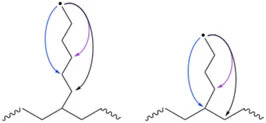
Available branch‐end radical hydrogen shift reactions based on ABL. On the left is ABL6, while on the right is ABL4. Purple is a 1,4‐hydrogen shift, blue is a 1,5‐hydrogen shift, and black is a 1,6‐hydrogen shift reaction.

**FIGURE 21:**
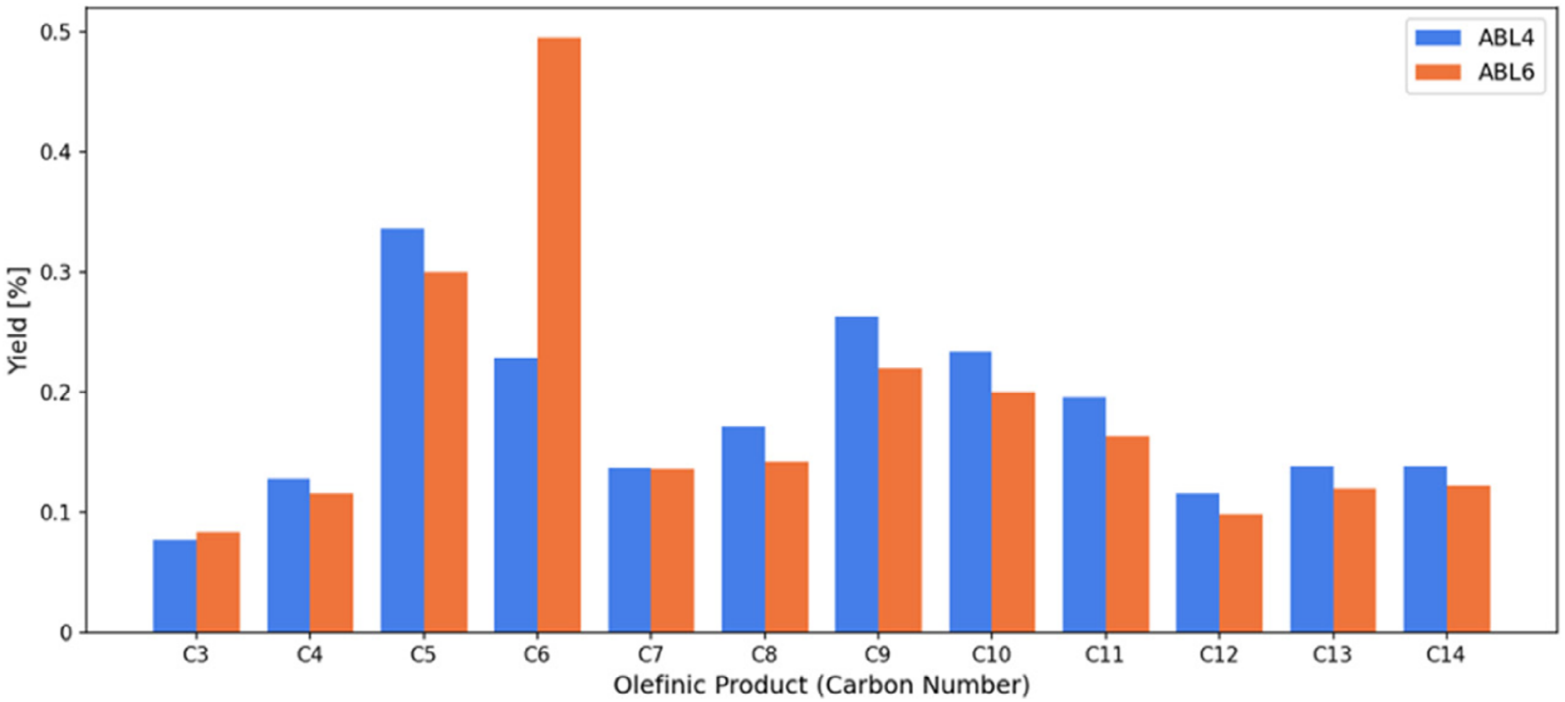
Comparison of low‐molecular weight product yields between ABL4 and ABL6 LLDPE samples after 4000 s at 673.15 K.

The performance of LLDPE with average branch lengths (ABL) corresponding to its different co‐monomers, such as α‐hexene (ABL = 4) and α‐octene (ABL = 6), was also analyzed in silico. Figure 17 illustrates the predicted LMWP yield and *M*_*n*_ decay trend of these different LLDPE samples. It can be observed that the *M*_*n*_ decay trend is very similar for both ABLs, while the low‐molecular‐weight product yields deviate slightly. The net‐rate analysis of chain‐fission reactions at branch points (Figure 18) shows that the rates are similar between the ABL4 and ABL6 systems. This is an expected result since they share the same kinetics and have the same concentration of initial branch points. However, if the branch‐backbone C—C bond breaks, the resulting low‐molecular‐weight radical species will have the same length as the ABL (four or six carbons long). Therefore, the difference in the net rate of hydrogen abstraction by low‐molecular weight radicals (Figure 19) is largely due to the difference in ABL lengths, as longer radicals abstract hydrogens more slowly than shorter radicals. Additionally, given the differences in branch length, branch end radicals undergoing a hydrogen shift reaction will result in a different suite of products. For example, in the ABL6 system, a 1,3‐, 1,4‐, or 1,5‐hydrogen shift will produce a C4, C5, or C6 branch radical. Alternatively, in the ABL4 system, those same shifts will result in a C4 branch radical, a tertiary branch point radical, or a mid‐chain radical on the polymer backbone (Figure 20). In turn, while ABL4 has higher yields of most products, ABL6 has a higher yield of 1‐hexene and propene (Figure 21). Propene is the result of a second *x*,*x* + 3 hydrogen shift from the C6 branch position followed by beta‐scission, while 1‐hexene is the result of beta‐scission at the C6 branch position. Both reaction paths are absent in the ABL4 system due to the shorter branch length. These observations suggest that average branch length plays a role in determining the available reaction pathways, the conversion, as well as the product distributions.

## 5 Conclusion

Insights into the mechanism of thermal pyrolysis of branched and non‐branched polyolefins are presented based on mechanistic analysis of PP, HDPE, and LLDPE pyrolysis reactions. HDPE, a non‐branched, linear polymer, is compared with polymers having different levels of branching. PP denotes the case of a heavily branched polymer with an extremely low branch length. An abundance of tertiary positions and methyl groups in this case results in faster decay rates and higher LMWP yield at a given reaction time. LLDPE is studied as the case with varying branch lengths at lower branch contents. The LLDPE model was newly developed and validated by comparison with experimental product distributions from flame ionization and time‐of‐flight spectrometer detectors (Py–GC×GC–FID/TOF–MS) temporal analysis. It was observed that radicals formed on the branches have a prominent role in determining the course of the reaction. Hydrogen abstraction from branches opens up reaction pathways that are unique to LLDPE. These pathways are competitive and could speed up/slow down the thermal degradation process. Various possible reaction pathways of these radicals were analyzed by changing the branch lengths and branch content. It was observed that average branch lengths have an impact on the reaction mechanism. For instance, LLDPE with ABL4 is predicted to produce a higher yield of oligomers than LLDPE with longer branches. These observations imply that it would be possible to tailor the LLDPE polymer to improve recyclability by adjusting its branch lengths and branch point density.

## Funding

This study was supported by The Dow Chemical Company.

## Conflicts of Interest

The authors declare no conflicts of interest.

## Supporting information

Supplementary Material

## Data Availability

The data that support the findings of this study are available from the corresponding author upon reasonable request.
