# Supplementary material for: Mechanistic Insights Into the Thermal Pyrolysis of Branched Polyolefins Using Kinetic Modeling
Source: ChemSusChem. 2026 Aug 30;19(17):e202502727. doi: 10.1002/cssc.202502727 (PMC13526906; doi:10.1002/cssc.202502727)
Supplement: Supplementary file 1 [file CSSC-19-e202502727-s001.pdf]

SUPPLEMENTARY INFORMATION

Mechanistic insights into the thermal pyrolysis of branched polyolefins using kinetic modeling

Aswathy K. Raghu[δ], Alexander K. Best[δ], Varaha Jayarama Krishna Jonnalagedda[^,+], Hilal Ezgi Toraman[+,^*], Paulami Majumdar[‡] and Linda J. Broadbelt[δ]*

[δ]*Department of Chemical and Biological Engineering, Northwestern University, 2145 Sheridan Road, Evanston, IL 60208, USA*
[+]*John and Willie Leone Family Department of Energy and Mineral Engineering, Penn State University, University Park, PA 16802, USA*
^*Department of Chemical Engineering, Penn State University, University Park, PA 16802, USA*
Institute of Energy and the Environment, Penn State University, University Park, PA 16802, USA

[‡]*Materials Discovery and Development, Core R&D, The Dow Chemical Company, Midland, MI*
+*Present address: Department of Chemical Engineering, Indian Institute of Technology Dharwad, Karnataka 580011, India*

**hzt5148@psu.edu*
*broadbelt@northwestern.edu

CALCULATION OF INITIAL CONDITIONS FOR THE MODEL

$$D0 = 0th\ moment\ of\ dead\ branched\ species\ class = \frac{Polymer\ mass\ density}{M_n}$$

$$D1 = 1st\ moment = D0 * \frac{M_n}{Monomer\ MW}$$

$$D2 = 2nd\ moment = D1 * \frac{M_w}{Monomer\ MW}$$

$$Br_{conc} = \frac{Num_{Br\_Pts\_1000C}}{1000} * \frac{Polymer\ mass\ density}{Monomer\ MW} * 2$$

$$Num_{Br\_Pts} = \frac{Br_{conc}}{D0}$$

Algebraic parameters in the LLDPE model

Number of branch points on the species (Num_{Br_Pts}) is an important parameter that determines the behavior of the LLDPE model. Reactions that form branched species as products will be favoured at high Num_{Br_Pts}, whereas reactions that form linear species will occur at lower Num_{Br_Pts}. All the possibilities for the reactions are incorporated into the model using algebraic parameters that multiply the rate equations. As the reaction proceeds, the LLDPE model transitions from a high branch content to a low branch content regime. Sigmoid functions are used to ensure smoothness during this transition. The formulas for the calculation of the algebraic variables are discussed here.

$$Num'_{Br\_Pts} = \frac{total\ branch\ concentration}{zeroth\ moment\ of\ all\ branched\ species\ classes}$$

$$Num_{Br\_Pts} = \frac{total\ branch\ concentration}{zeroth\ moment\ of\ all\ branched\ species\ classes} * \frac{1}{1 + \mathrm{e}^{-(4\ Num'_{Br\_Pts} - 5)}}$$

As the reaction nears the end, the branch concentration might get over-predicted because of the shrinkage in volume. $Num'_{Br_Pts}$is used to ensure that Num_{Br_Pts}remains low even when it is incorrectly predicted as high.

$$F_{p_1} = \frac{Concentration\ of\ branched\ species\ saturated\ ends}{Concentration\ of\ branched\ species\ (saturated + unstaurated)\ ends}$$

$$F_{P_1} = \frac{Concentration\ of\ branched\ species\ unsaturated\ ends}{Concentration\ of\ branched\ species\ (saturated + unstaurated)\ ends}$$

These variables are used to differentiate the products based on the end types (saturated/unsaturated) and is accordingly multiplied to the algebraic parameter.

$$F_{end\ chain} = \frac{2}{Num_{Br\_Pts} + 1}$$

$$F_{mid\ chain} = 1 - F_{end\ chain}$$

The reactions in the model where a variety of products are likely to form and the corresponding algebraic parameters are described below. The algebraic parameters are used whenever required, and are indicated in blue color in the figures.

(a) Chain fission & Recombination

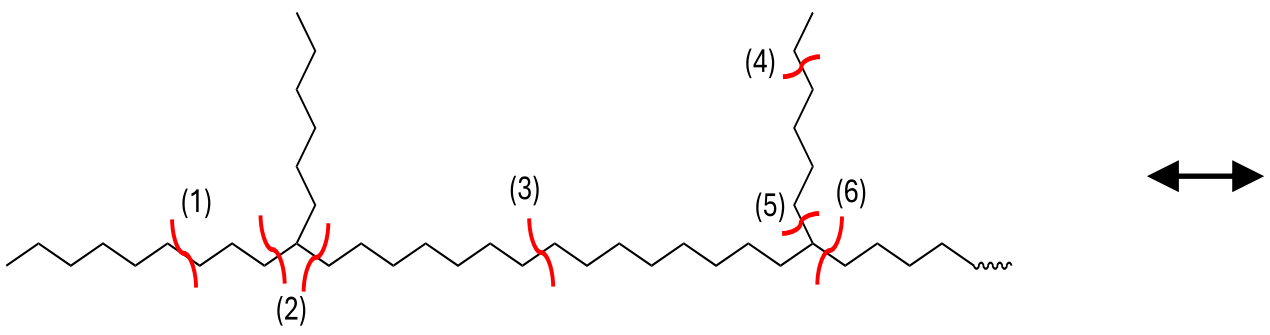

(1)

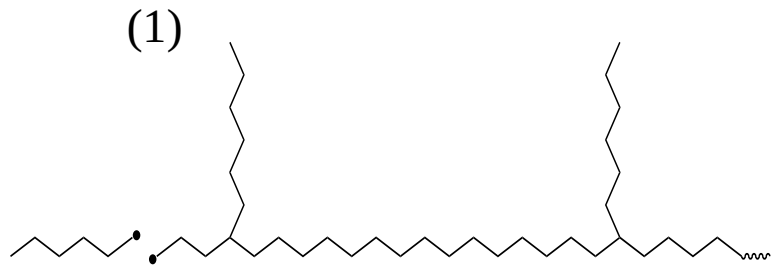

Linear end-radical + Branched end-radical

$F_{end\ chain}$

(2)

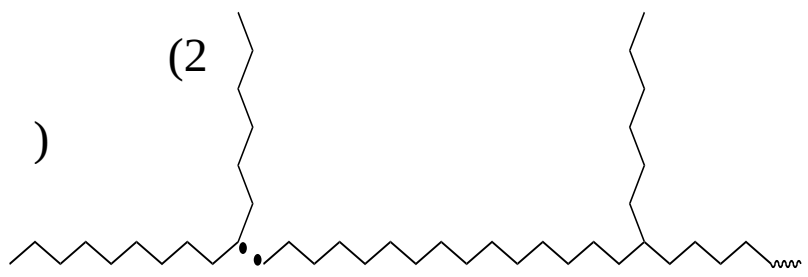

Linear end-radical + Branched mid-radical or Linear mid-radical + Branched end-radical

(3)

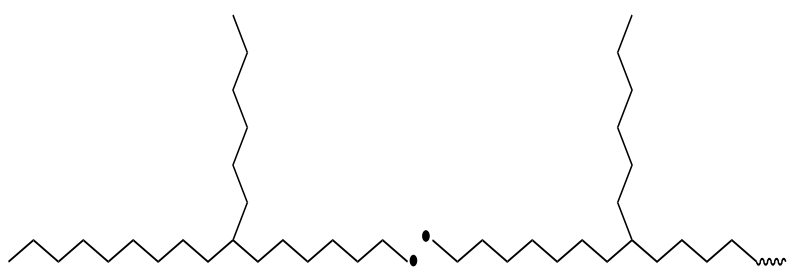

Branched end-radical + Branched end-radical

$F_{mid\ chain}$

(4)

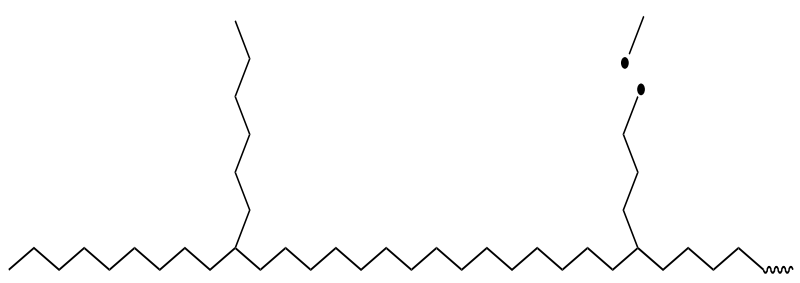

Small radical + Branched end-radical

$Num_{Br\_Pts} \times \frac{all\ specific\ branches\ (sat/\ unsat\ end)\ with\ length\ >=\ speci}{total\ branches}$

(5)

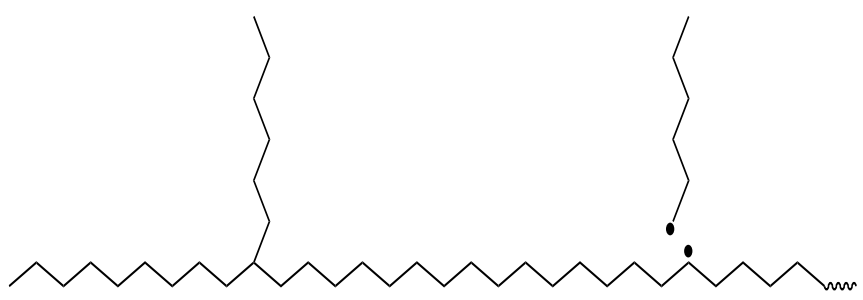

Small radical + Branched mid-radical

$Num_{Br\_Pts} \times \frac{specific\ branches\ (sat/\ unsat\ end)\ with\ specific\ leng}{total\ branches}$

(6)

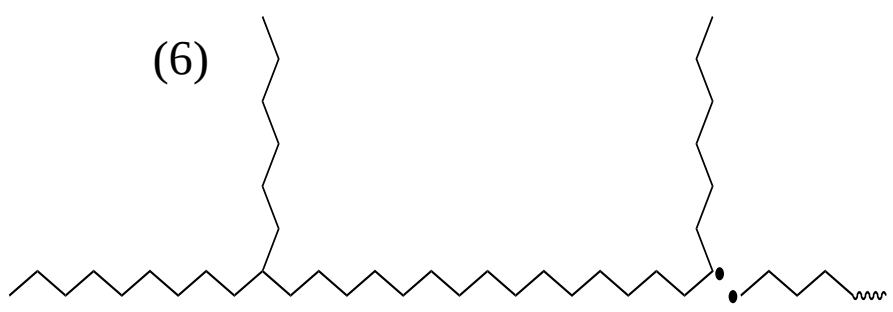

Branched mid-radical + Branched end-radical

$2 \times Num_{Br\_Pts} \times \frac{branches\ with\ specific\ length\ (\#C)}{total\ branches}$

(b) Hydrogen abstraction (chain transfer)

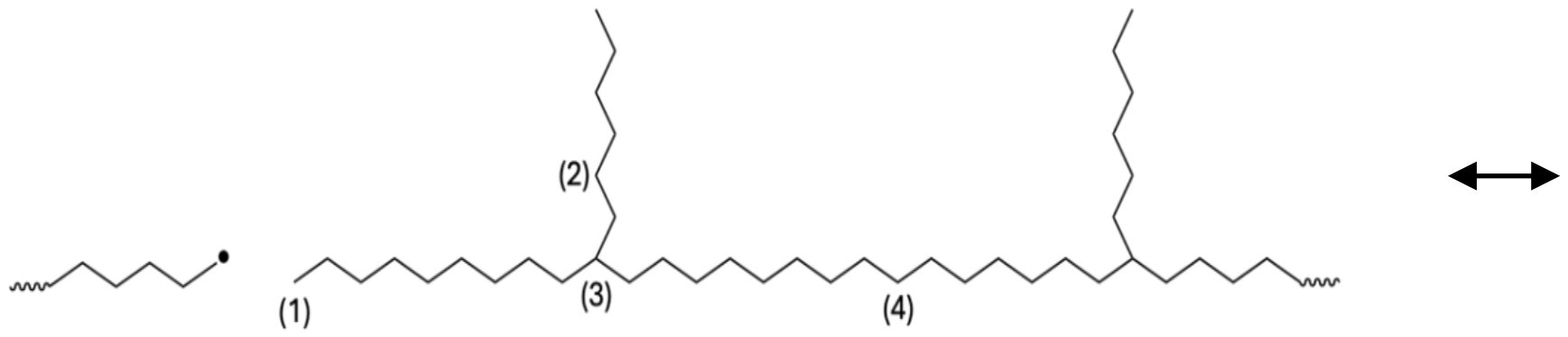

(1)

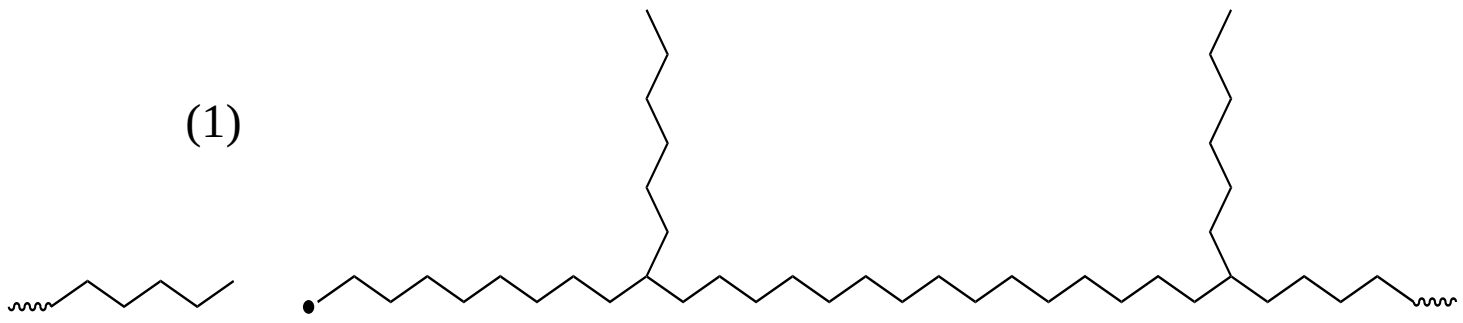

Branched end-radical

$2 \times F_{p_1}$

(2

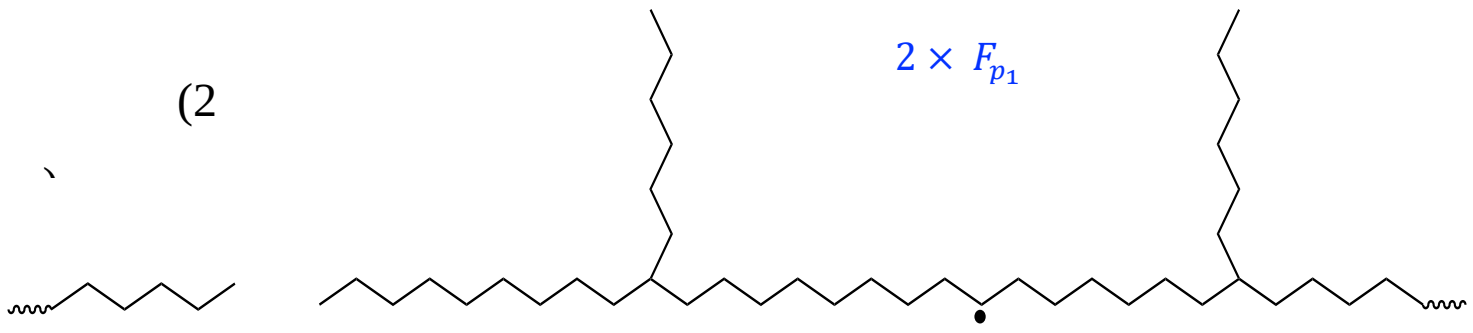

Branched mid-

(3)

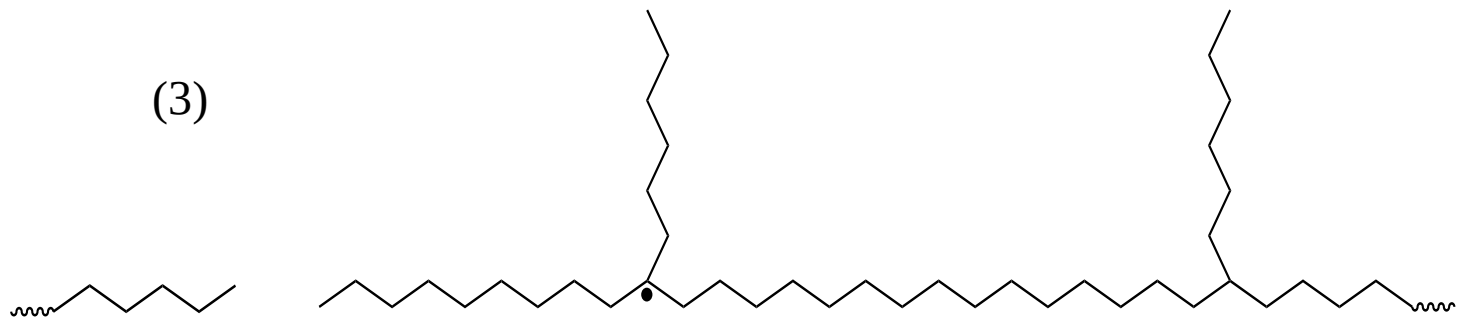

Branched tertiary radical

Num_{Br_Pts}

(4)

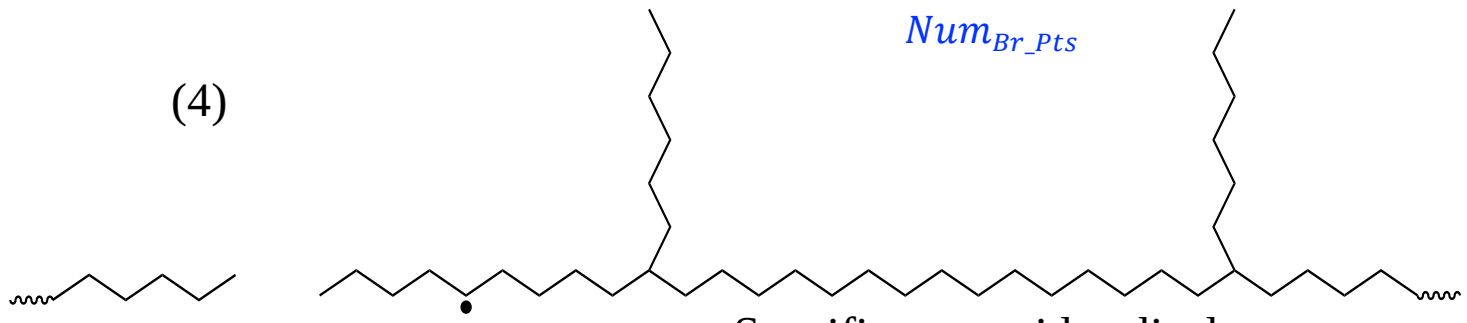

Specific mid-radical on backbone

$2 \times F_{p_1}$ $(or\ F_{P_1}\ if\ dienes\ are\ considered)$

(5)

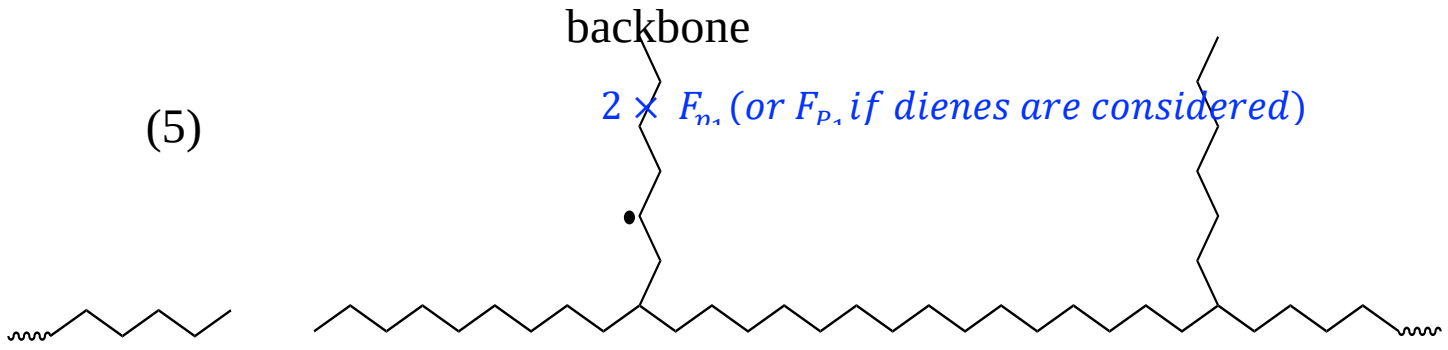

Specific mid-radical on branch

$$Num_{Br\_Pts} \times \frac{all\ specific\ branches\ (sat\ ends) with\ length\ >=\ specific\ length\ (\#C)}{total\ branches}$$

(6)

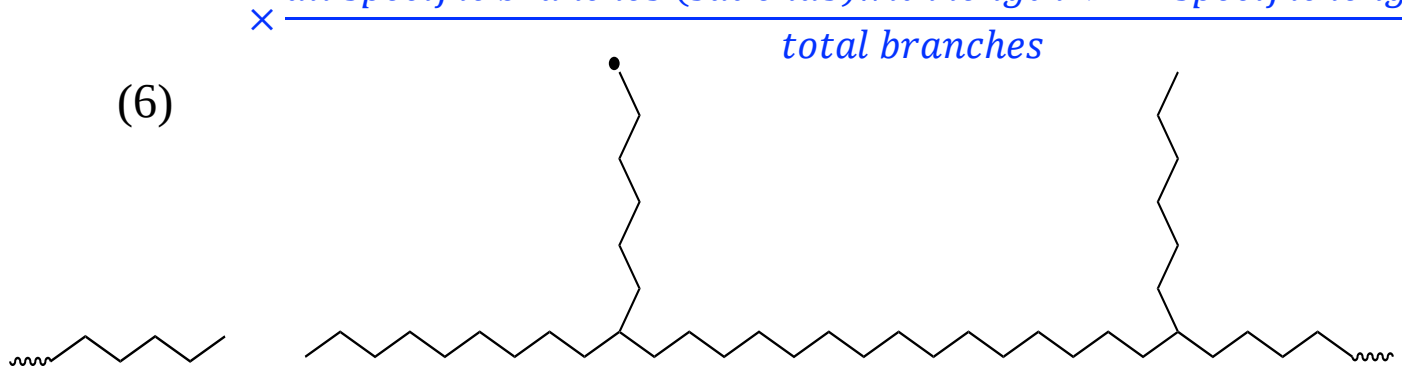

End-radical on branch

Num_{Br_Pts}

(c) Hydrogen shifts

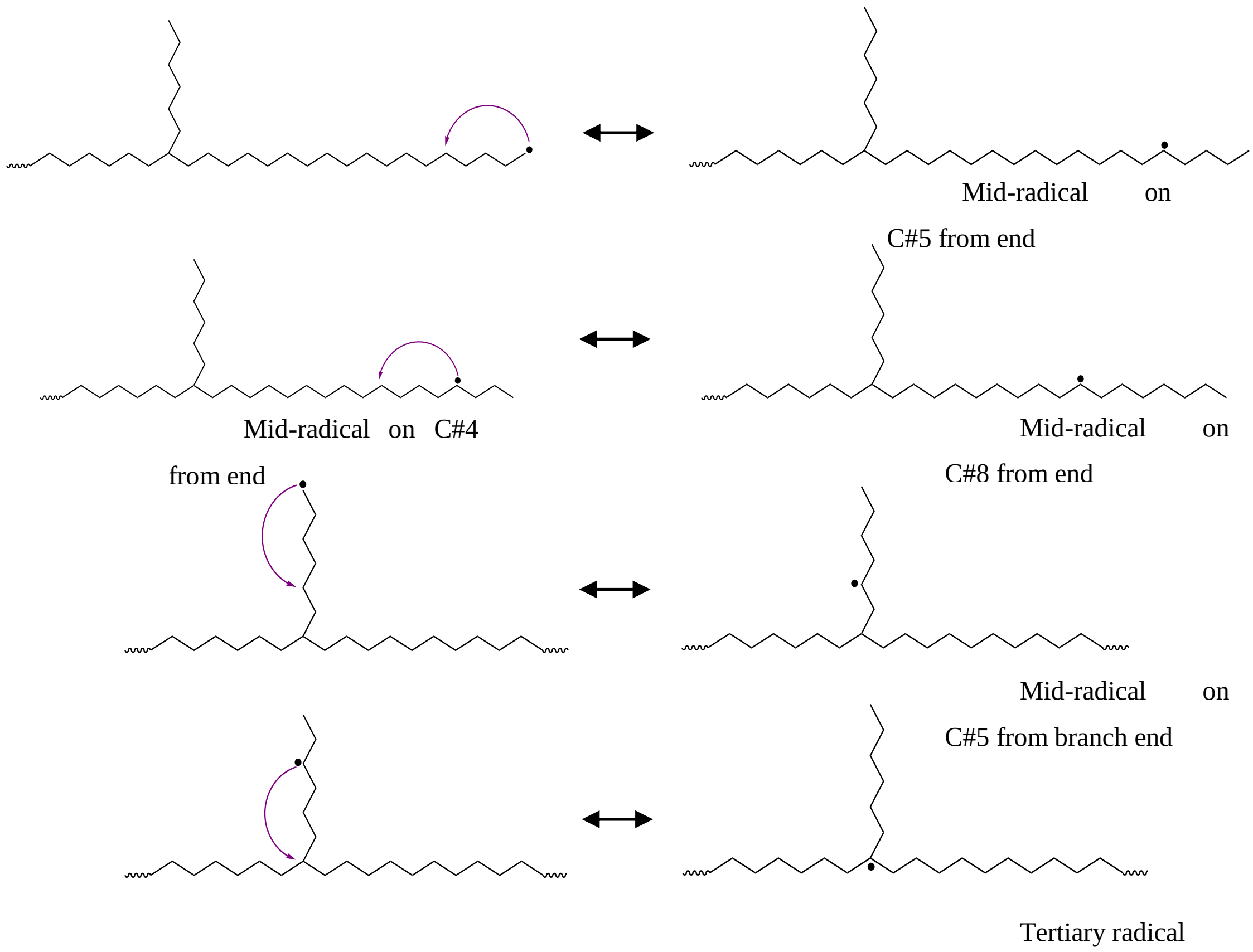

Algebraic multipliers are not necessary in these cases because the radical species classes are separated based on their position.

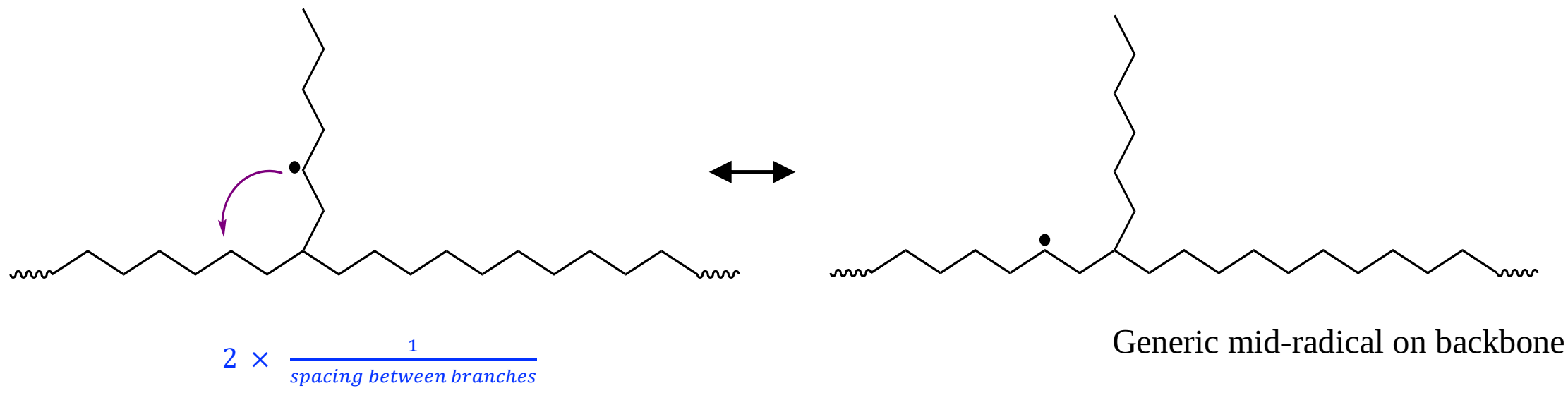

An algebraic parameter is used in the reverse reaction in the above case because the generic mid-radical class is the reactant, and there are only two positions, each on either side of the branch, from where this shift can happen.

(d) Mid-chain beta

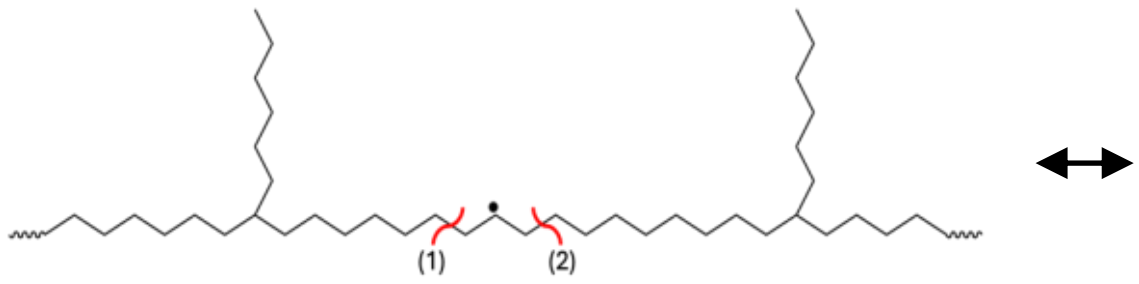

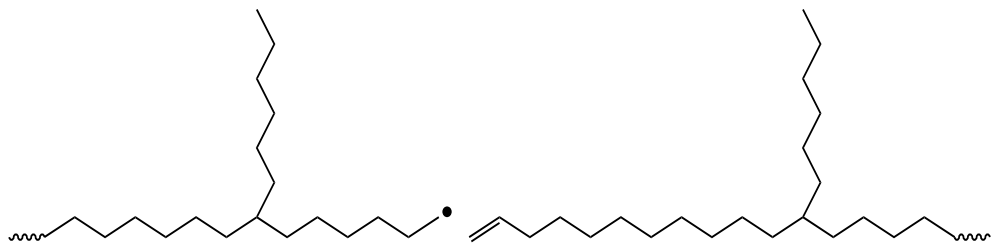

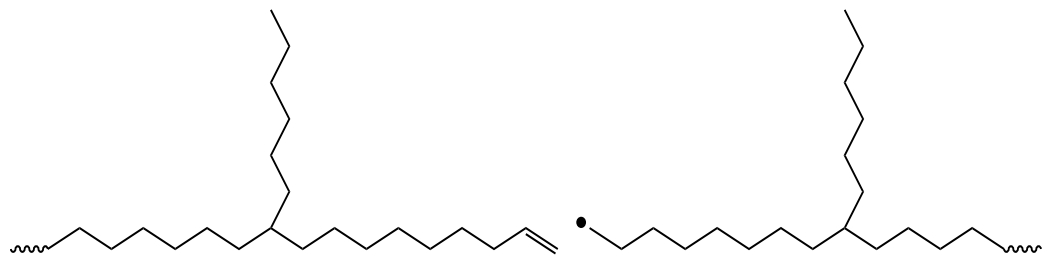

Branched end-radical + Branched dead species

$F_{mid\ chain} \times (1 -$

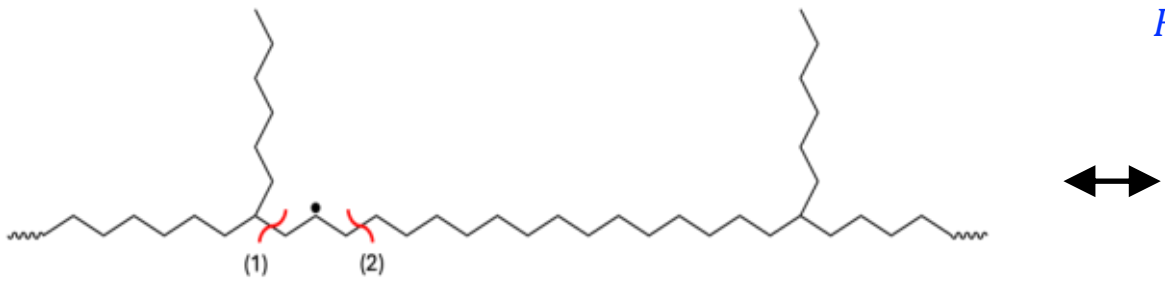

Branched mid-radical + Branched dead species

$F_{mid\ chain} \times \frac{1}{spacing\ between\ branches} \times \frac{branches\ with\ specific\ length\ (\#C)}{total\ branches}$

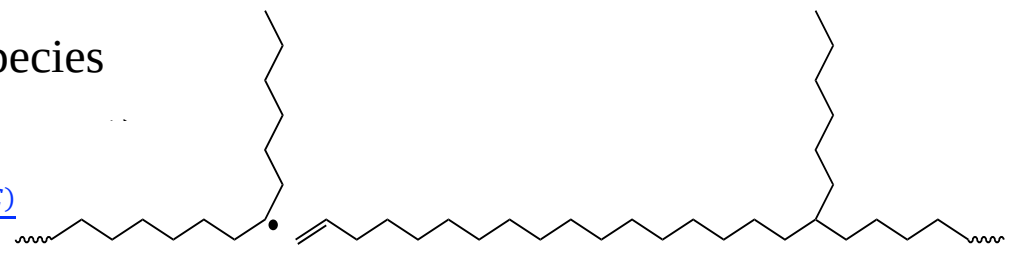

Branched end-radical + Branched dead species

$F_{mid\ chain} \times \frac{1}{spacing\ between\ branches}$

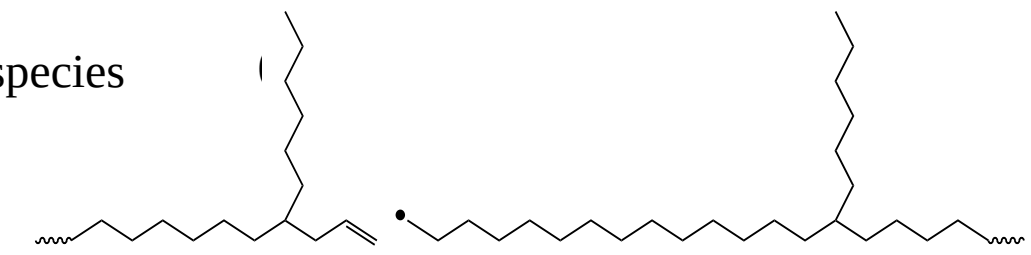

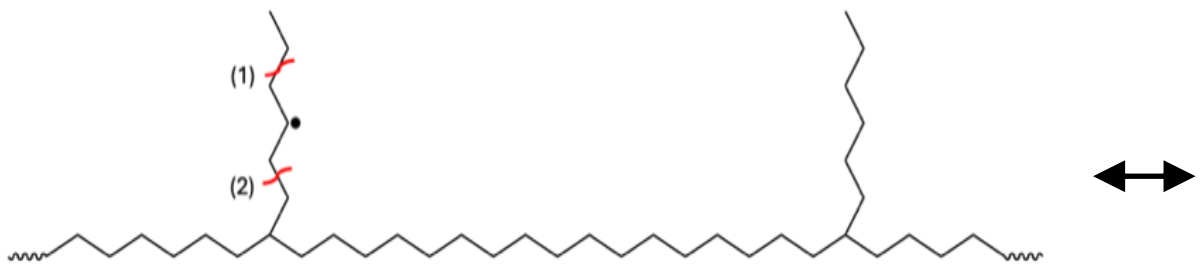

Small end-radical + Branched dead species

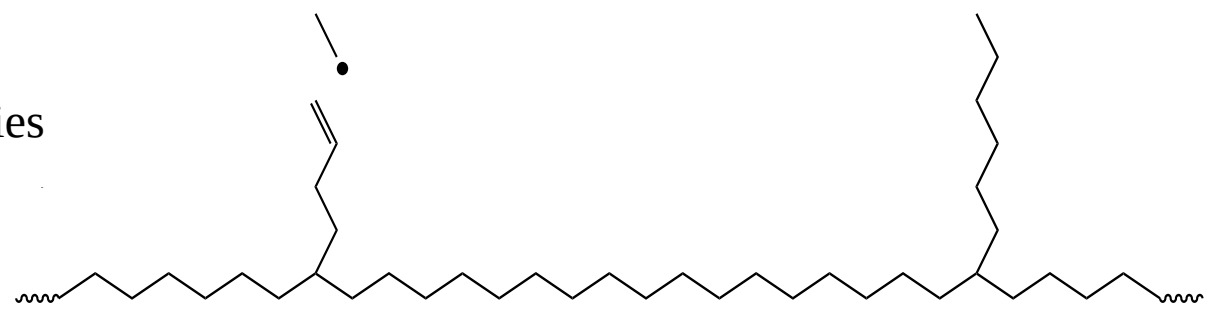

Branched end-radical + Small dead species

$1 - \frac{branches\ with\ specific\ length\ (\#C)}{all\ branches\ with\ length\ >=\ specific\ length\ (\#C)}$

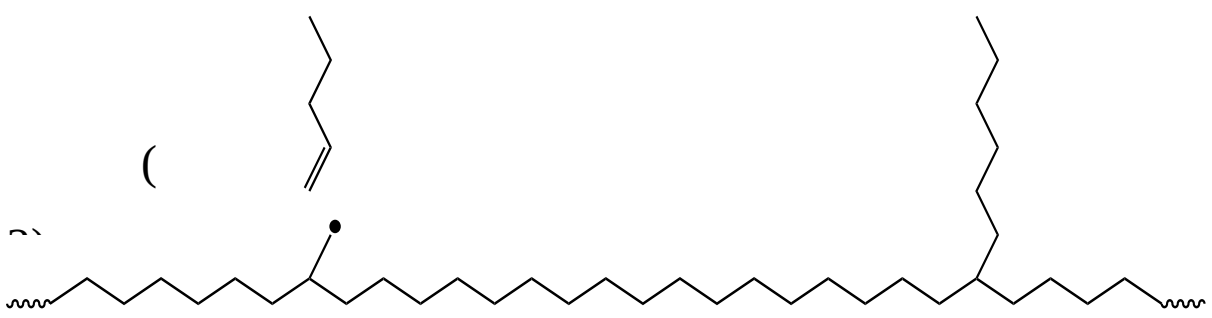

(d) Mid-chain beta scission

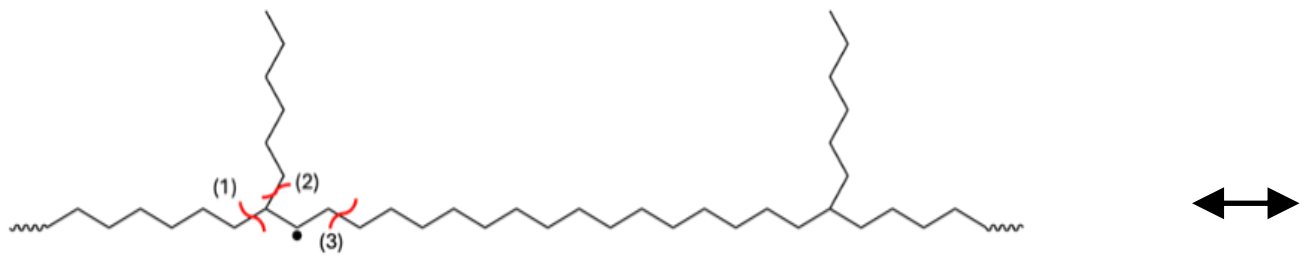

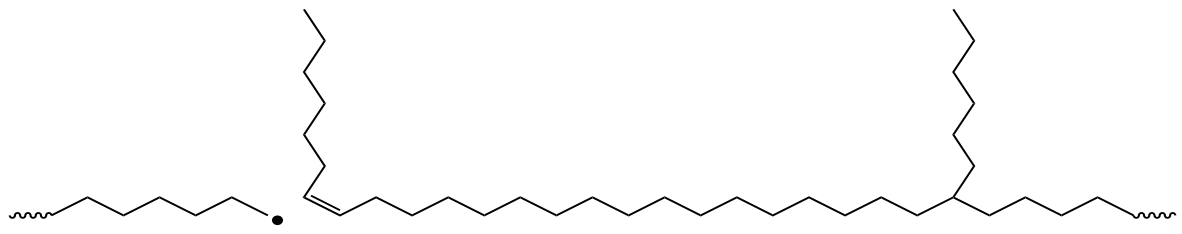

Branched end-radical + Branched dead species

$$F_{mid\ chain} \times \frac{1}{spacing\ between\ branches}$$

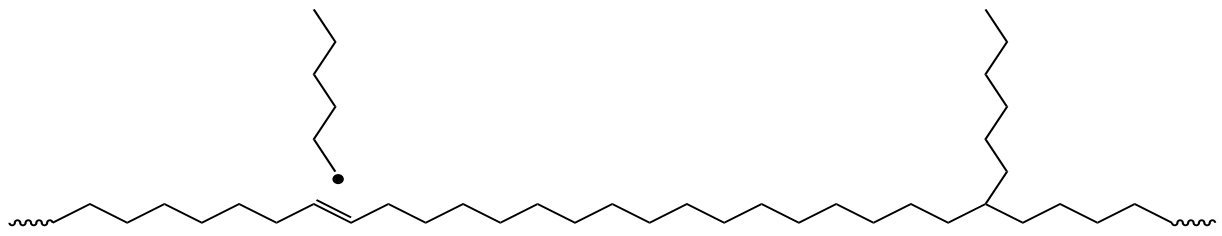

Small radical + Branched dead species

$$F_{mid\ chain} \times \frac{1}{spacing\ between\ branches} \times \frac{branches\ with\ specific\ length\ (\#C)}{total\ branches}$$

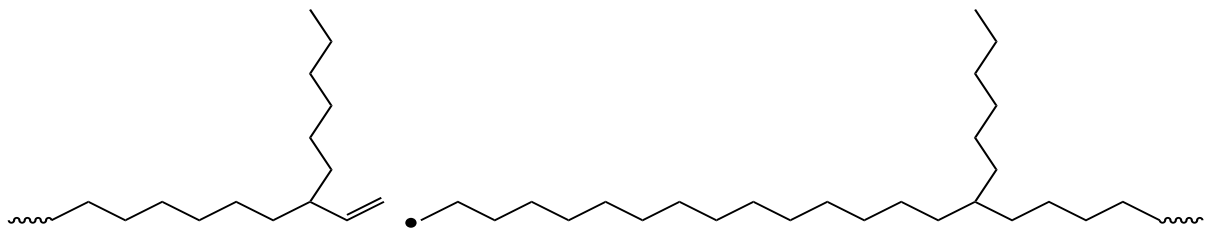

Branched end-radical + Branched dead species

$$F_{mid\ chain} \times \frac{1}{spacing\ between\ branches}$$

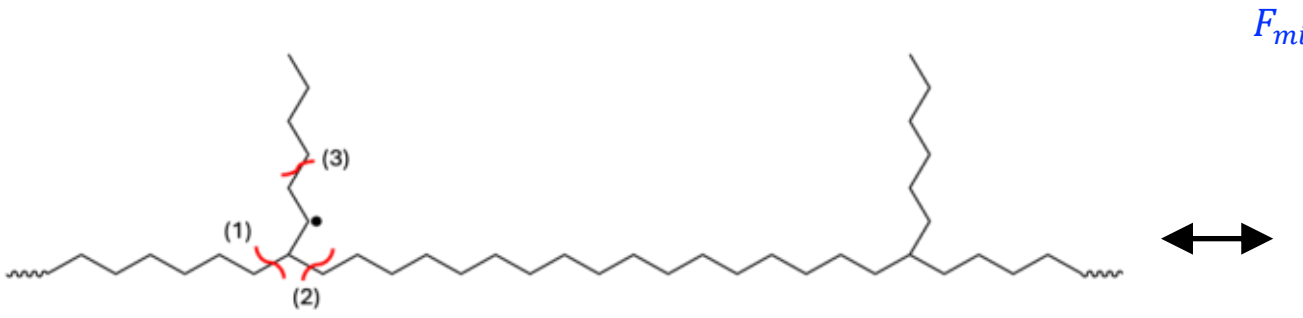

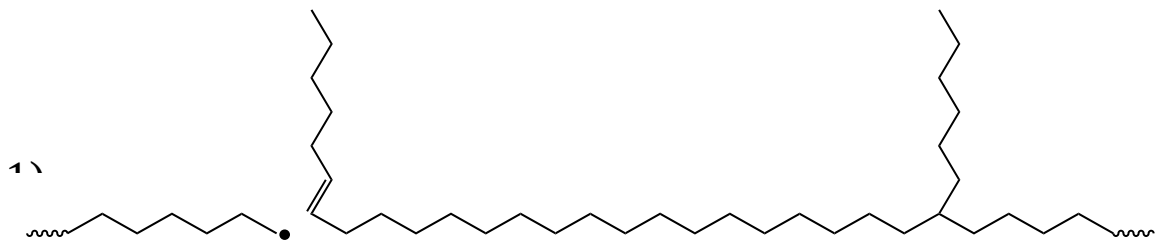

Branched end-radical + Branched dead species

$$\frac{Num_{Br\_Pts}}{Num_{Br\_Pts}+2} \times \frac{branches\ with\ specific\ length\ (\#C)}{all\ branches\ with\ length\ >=\ specific\ length\ (\#C)}$$

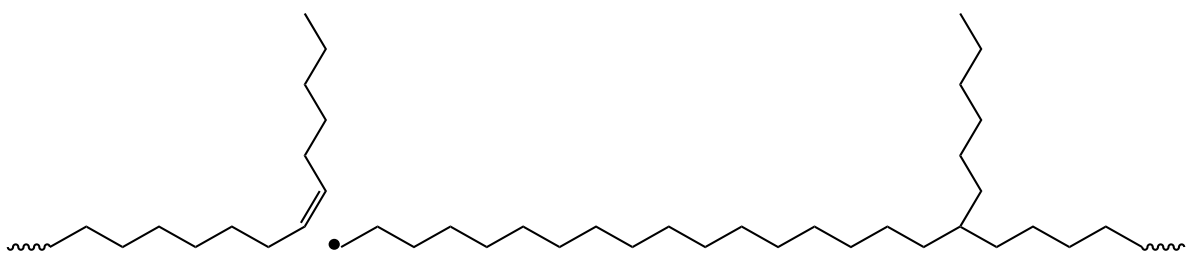

Branched end-radical + Branched dead species

$$\frac{Num_{Br\_Pts}}{Num_{Br\_Pts}+2} \times \frac{branches\ with\ specific\ length\ (\#C)}{all\ branches\ with\ length\ >=\ specific\ length\ (\#C)}$$

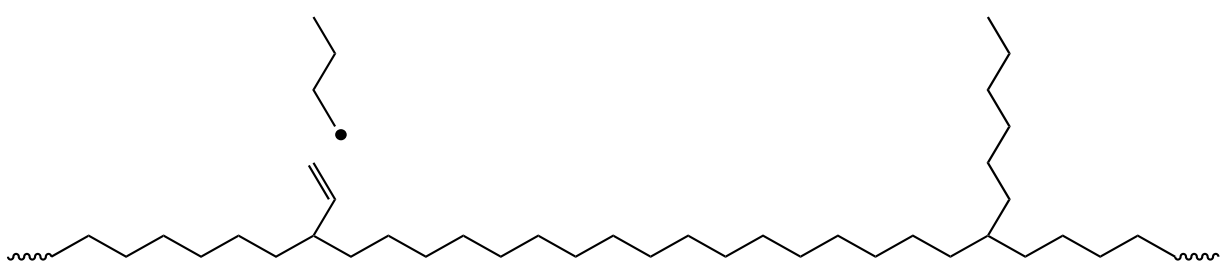

Small end-radical + Branched dead species

(d) Mid-chain beta scission

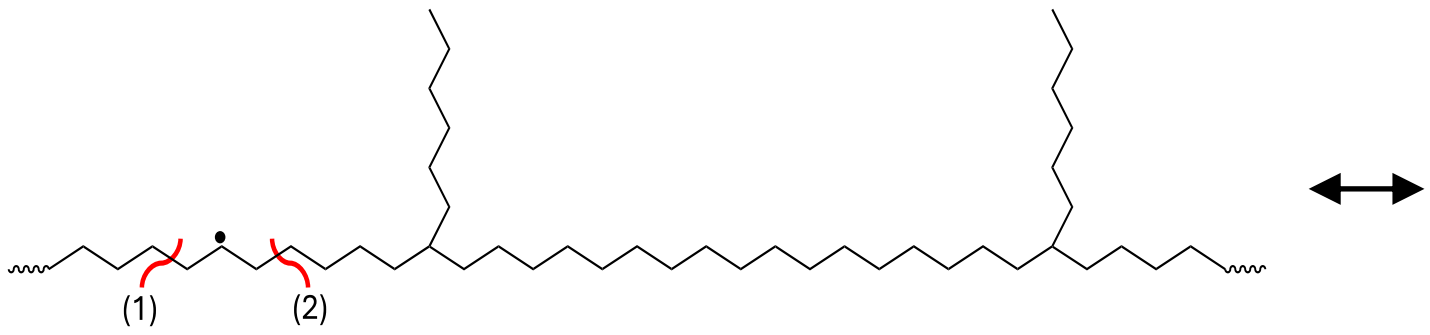

Linear end-radical + Branched dead species

$F_{end\ chain} \times (1 - \frac{4}{spacing\ between\ branches})$

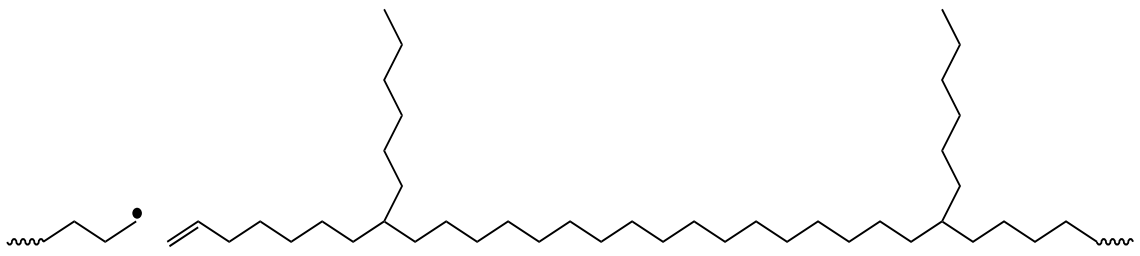

Linear dead species + Branched end-radical

$F_{end\ chain} \times (1 - \frac{4}{spacing\ between\ branches})$

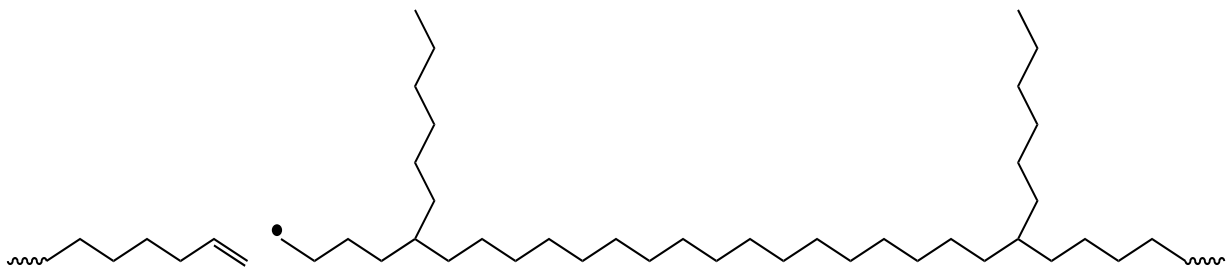

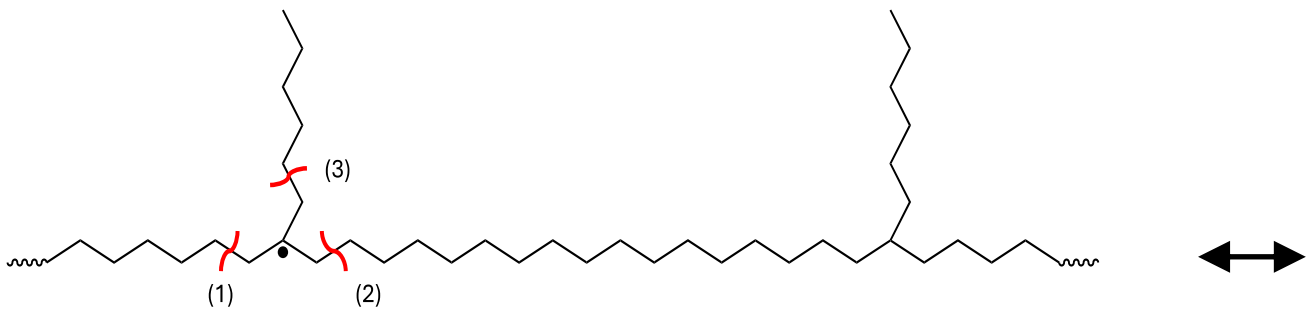

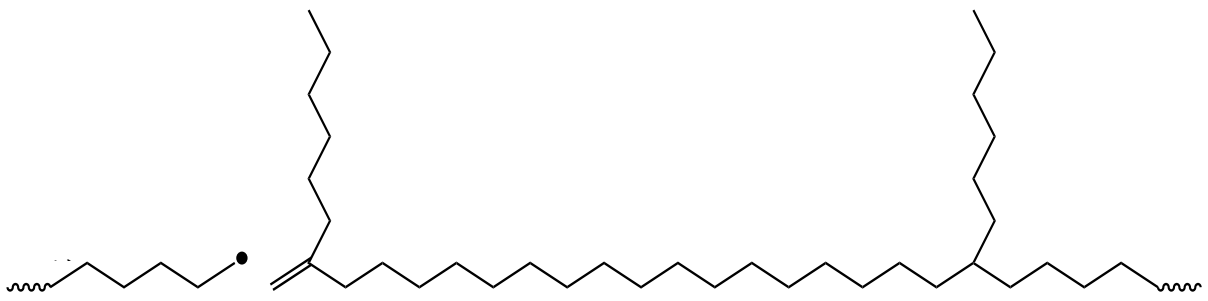

Branched end-radical + Branched dead species

$F_{mid\ chain}$

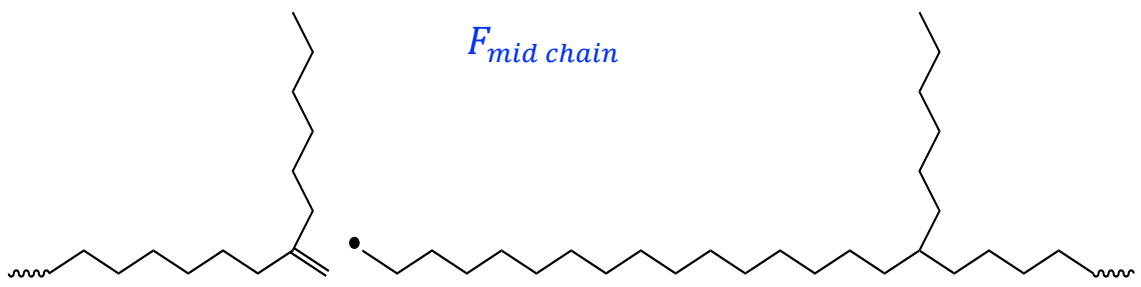

Branched end-radical + Branched dead species

$F_{mid\ chain}$

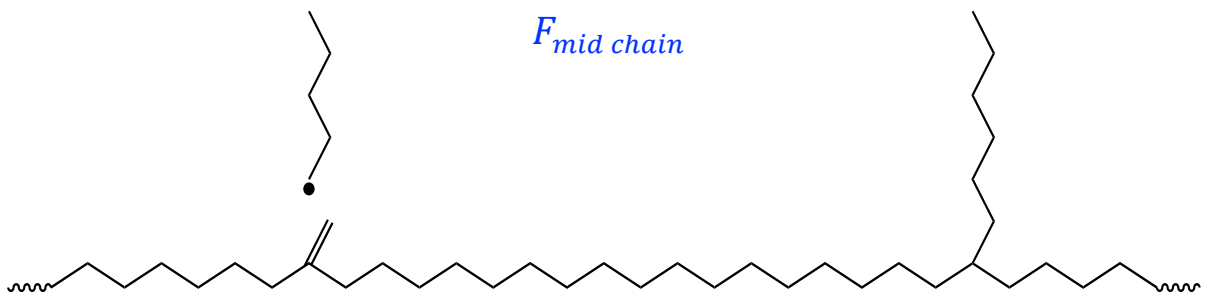

Small radical + Branched dead species

$\frac{branches\ with\ specific\ length\ (\#C)}{total\ branches}$

(e) End-chain beta scission

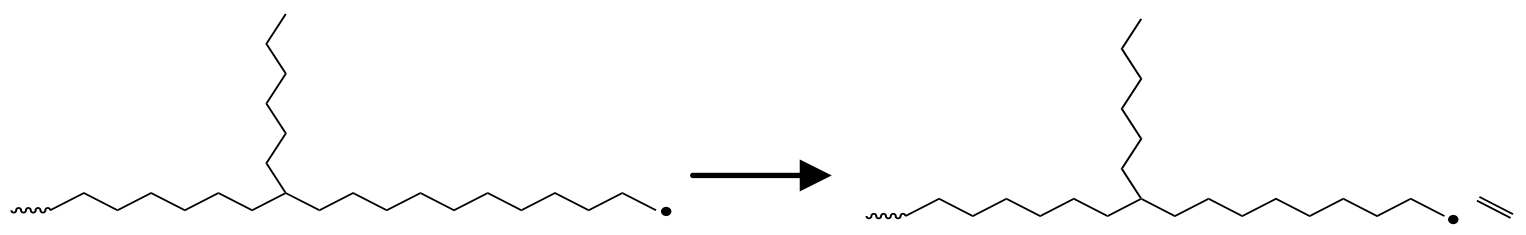

Branched end-radical + Monomer

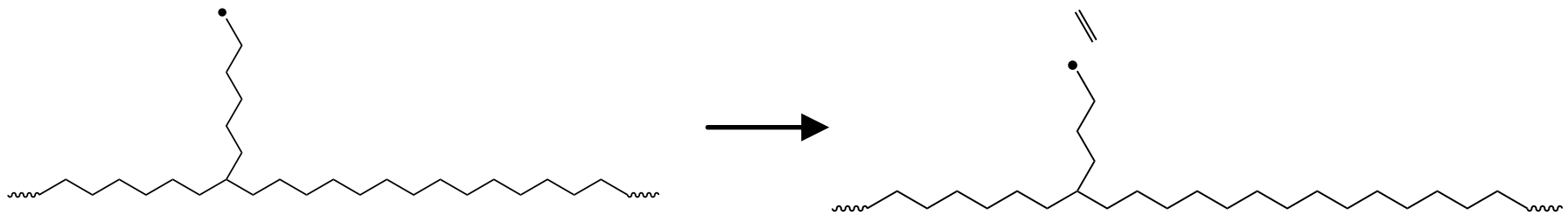

Branched end-radical (on branch) + Monomer

$$1-\frac{branches\ with\ length\ C\#2}{total\ branches}$$

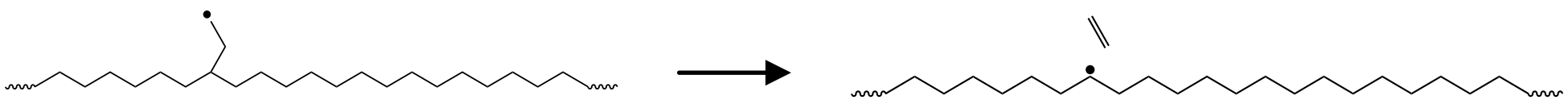

Branched mid-radical + Monomer

$$\frac{branches\ with\ length\ C\#2}{total\ branches}$$

(f) Disproportionation

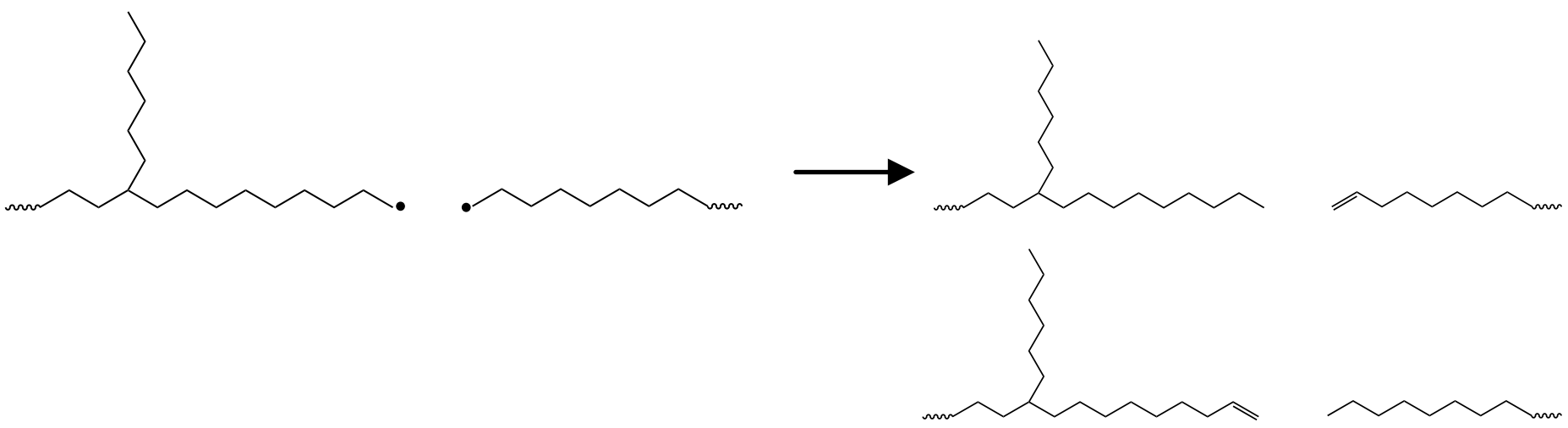

Dead species with saturated and unsaturated ends

Disproportionation reactions are included for all pairs of radicals.

Experimental Results Used for LLDPE Model Validation

Table S1. The yield (wt.%) of dienes from LLDPE pyrolysis based on carbon number at 400 °C and different pyrolysis times.

**Carbon Number**	**2 min**	**3 min**	**4 min**	**5 min_Avg**	**Std**
11	0.0002	0.0004	0.0007	0.0008	0.0001
12	0.0003	0.0005	0.0006	0.0008	0.0001
13	0.0004	0.0005	0.0009	0.001	0.0001
14	0.0004	0.0005	0.0009	0.001	0.0001
15	0.0003	0.0006	0.0008	0.001	0.0001
16	0.0005	0.0008	0.0011	0.001	0.0002
17	0.0006	0.0007	0.0011	0.002	0.0001
18	0.0008	0.0009	0.0016	0.002	0.0002
19	0.0008	0.0011	0.0014	0.002	0.0001
20	0.0009	0.001	0.0013	0.002	0.0001
21	0.0009	0.0014	0.0016	0.003	0.0001
22	0.0006	0.0008	0.0012	0.002	0.0001
23	0.0009	0.001	0.0014	0.002	0.0002
24	0.0003	0.0006	0.0008	0.001	0.0001
25	0.0005	0.0008	0.0012	0.002	0.0001
26	0.0008	0.0011	0.0014	0.002	0.0001
27	0.0009	0.0012	0.0017	0.002	0.0002
28	0.0008	0.0012	0.0018	0.002	0.0001

Table S2. The yield (wt.%) of olefins from LLDPE pyrolysis based on carbon number at 400 °C and different pyrolysis times.

Carbon Number	1 min	2 min	3 min	4 min	5 min_Avg	std
4	0.0009	0.0014	0.0012	0.0014	0.0032	0.0006
5	0.0082	0.0117	0.013	0.0148	0.0181	0.0017
6	0.0309	0.0494	0.0597	0.0871	0.1078	0.0072
7	0.0037	0.0062	0.0071	0.0092	0.0141	0.0004

8	0.0045	0.0061	0.0076	0.0094	0.0147	0.0006
9	0.003	0.0041	0.006	0.0067	0.0114	0.0005
10	0.004	0.0065	0.0078	0.0098	0.0153	0.0002
11	0.0033	0.0057	0.0072	0.0089	0.014	0.0001
12	0.0036	0.0064	0.0079	0.0103	0.0137	0.0012
13	0.0024	0.0046	0.006	0.0094	0.0109	0.0002
14	0.0037	0.0053	0.0069	0.0097	0.0125	0.0004
15	0.0026	0.0031	0.0056	0.0077	0.0112	0.001
16	0.0035	0.0058	0.0073	0.01	0.0123	0.0016
17	0.0018	0.0022	0.0043	0.0058	0.0072	0.0006
18	0.0039	0.0061	0.0073	0.0079	0.0116	0.0002
19	0.0015	0.0038	0.0051	0.0059	0.0074	0.0004
20	0.0025	0.004	0.005	0.0061	0.0082	0.0006
21	0.0024	0.0038	0.0044	0.0056	0.008	0.0009
22	0.0021	0.004	0.0056	0.0065	0.0103	0.0003
23	0.0013	0.0026	0.0037	0.0049	0.0071	0.0002
24	0.002	0.0033	0.0042	0.005	0.0075	0.0006
25	0.0009	0.0023	0.0031	0.0048	0.006	0.0004
26	0.0012	0.0041	0.0051	0.0063	0.0089	0.0008
27	0.0015	0.0024	0.003	0.0043	0.0062	0.0006
28	0.0035	0.0075	0.0095	0.012	0.0173	0.0014

Table S3. The yield (wt.%) of paraffins based on carbon number at 400 °C and different pyrolysis times.

**Carbon Number**	**1 min**	**2 min**	**3 min**	**4 min**	**5 min_Avg**	**std**
5	0.0268	0.0489	0.0582	0.0527	0.0871	0.0169
6	0.0133	0.0231	0.0291	0.0415	0.0508	0.0018
7	0.0027	0.0048	0.0062	0.0074	0.0107	0.0012
8	0.0	0.0	0.0	0.0	0.011	0.0

	029	048	059	071	3	009
9	0.0034	0.0036	0.007	0.0084	0.0118	0.0013
10	0.0016	0.0026	0.0034	0.0047	0.0066	0.0004
11	0.002	0.0033	0.0043	0.0057	0.0075	0.0004
12	0.0022	0.0035	0.0043	0.0057	0.0077	0.0006
13	0.0019	0.0031	0.0038	0.0054	0.0073	0.0004
14	0.0016	0.0026	0.0031	0.0051	0.0067	0.0003
15	0.0016	0.003	0.0039	0.0055	0.0079	0.0005
16	0.002	0.0027	0.0035	0.0049	0.0071	0.0004
17	0.0014	0.0025	0.0033	0.005	0.0069	0.0004
18	0.0013	0.0025	0.0033	0.0041	0.0059	0.0003
19	0.0016	0.0023	0.003	0.0042	0.0057	0.0002
20	0.0009	0.0022	0.0028	0.0037	0.0055	0.0004
21	0.0014	0.0025	0.0029	0.0041	0.006	0.0002
22	0.0012	0.0021	0.0027	0.0034	0.0047	0.0004
23	0.0013	0.0021	0.0028	0.0039	0.0057	0.0004
24	0.0015	0.0028	0.0034	0.0037	0.0061	0.0006
25	0.0016	0.003	0.0039	0.0043	0.0049	0.0004
26	0.0028	0.0041	0.0054	0.0062	0.007	0.0004
27	0.0017	0.0032	0.0037	0.0054	0.0062	0.0003
28	0.0016	0.0028	0.0032	0.004	0.0063	0.0006

Table S4. The yield (wt.%) of diolefins from LLDPE pyrolysis based on carbon number at 430 °C and different pyrolysis times.

**Carbon Number**	**1 min**	**2 min_Avg**	**std**	**3 min**	**4 min**	**5 min_Avg**	**Std**
9	0.0013	0.0021	0.0002	0.0042	0.005	0.0059	0.0005
10	0.0012	0.0018	0.0003	0.0039	0.0044	0.0053	0.0001
11	0.0011	0.0018	0.0001	0.0035	0.0041	0.0055	0.0002
12	0.0018	0.0019	0.0003	0.0039	0.0047	0.006	0.0002
13	0.0019	0.0023	0.0004	0.0047	0.0058	0.0073	0
14	0.0022	0.0026	0.0002	0.0051	0.0059	0.0077	0
15	0.001	0.0029	0.0003	0.0056	0.0066	0.0085	0.0001
16	0.0018	0.0033	0.0004	0.0059	0.0075	0.0093	0.0001
17	0.0021	0.0038	0.0002	0.0077	0.0106	0.0117	0.0015
18	0.0021	0.0036	0.0007	0.0074	0.0089	0.0111	0.0002
19	0.0028	0.0044	0.0008	0.0089	0.011	0.0132	0.0015
20	0.0023	0.0041	0.0005	0.0088	0.0104	0.0135	0.0005
21	0.0025	0.0045	0.0006	0.0092	0.0114	0.0142	0.0001
22	0.0025	0.0044	0.0006	0.0079	0.0107	0.0133	0.0001
23	0.0025	0.0045	0.0006	0.0093	0.0118	0.0147	0
24	0.0026	0.0043	0.0006	0.0088	0.0112	0.0147	0.0007
25	0.0023	0.0046	0.0003	0.0089	0.0109	0.0146	0.0003
26	0.002	0.004	0.0006	0.0078	0.0113	0.014	0.0005
27	0.0022	0.0049	0.0006	0.0091	0.0148	0.015	0.0002
28	0.002	0.0037	0.0006	0.0079	0.0096	0.012	0.0002

Table S5. The yield (wt.%) of olefins from LLDPE pyrolysis based on carbon number at 430 °C and different pyrolysis times.

**Carbon Number**	**1 min**	**2 min_Avg**	**std**	**3 min**	**4 min**	**5 min_Avg**	**Std**
4	0.0018	0.0042	0.0012	0.0089	0.0074	0.0179	0.0048
5	0.0884	0.1513	0.0036	0.2317	0.2902	0.2841	0.0227
6	0.1298	0.224	0.0248	0.3123	0.3812	0.4416	0.0194
7	0.0222	0.0372	0.0043	0.054	0.0724	0.0803	0.0012
8	0.0198	0.0367	0.0024	0.048	0.0522	0.0639	0.0011
9	0.0142	0.0357	0.0033	0.0442	0.0594	0.0728	0.0019
10	0.0201	0.0335	0.0021	0.0621	0.0789	0.091	0.0027
11	0.0168	0.0258	0.001	0.0429	0.0536	0.0626	0.0009
12	0.0216	0.0273	0.0015	0.0442	0.0533	0.0631	0.0003
13	0.0107	0.0187	0.0006	0.0309	0.0362	0.045	0.0012
14	0.0153	0.0253	0.0018	0.0397	0.0446	0.0587	0.0014
15	0.0144	0.025	0.0011	0.0406	0.0473	0.0618	0.0008
16	0.0173	0.0225	0.0016	0.0319	0.0366	0.0492	0.0008
17	0.0119	0.0171	0.0012	0.0294	0.0345	0.0448	0.0005
18	0.0133	0.0185	0.0022	0.035	0.0415	0.0514	0.0004
19	0.0109	0.0146	0.0021	0.0304	0.0374	0.0464	0.0005
20	0.0099	0.0164	0.0014	0.0315	0.0393	0.0484	0.0009
21	0.0079	0.0128	0.0021	0.0258	0.031	0.0394	0.0001
22	0.0061	0.014	0.0022	0.0276	0.0329	0.0419	0.0002
23	0.0079	0.0147	0.002	0.0305	0.0362	0.0473	0.0008
24	0.0088	0.0138	0.002	0.0271	0.0291	0.0415	0.0001
25	0.0053	0.0115	0.0014	0.0246	0.0302	0.039	0.0003
26	0.0071	0.0133	0.0013	0.0257	0.0308	0.0406	0.0004
27	0.0041	0.0109	0.0013	0.0245	0.0288	0.0378	0.0004
28	0.0067	0.0121	0.0014	0.0252	0.0303	0.0378	0.0006

Table S6. The yield (wt.%) of paraffins from LLDPE pyrolysis based on carbon number at 430 °C and different pyrolysis times.

Carbon Number	1 min	2 min_Avg	std	3 min	4 min	5 min_Avg	Std
5	0.0065	0.0085	0.003	0.0158	0.0126	0.0152	0.0031
6	0.0657	0.1114	0.018	0.1412	0.1864	0.2119	0.0001
7	0.0164	0.0285	0.0023	0.0424	0.0555	0.0625	0.0007
8	0.0143	0.0273	0.004	0.0373	0.0429	0.0505	0.0008
9	0.0144	0.0196	0.0011	0.0303	0.0397	0.0486	0.0007
10	0.0077	0.0123	0.0007	0.0212	0.0247	0.0305	0.0001
11	0.0098	0.0156	0.0008	0.0261	0.0299	0.0371	0.0001
12	0.0096	0.0152	0.0009	0.025	0.0299	0.0365	0.0004
13	0.0084	0.0148	0.0006	0.0234	0.0257	0.0338	0.0012
14	0.0074	0.0134	0.0005	0.02	0.023	0.03	0.0011
15	0.0082	0.0156	0.0009	0.0229	0.0255	0.0334	0.0011
16	0.008	0.0129	0.0009	0.0208	0.0247	0.0308	0.0009
17	0.0071	0.0127	0.0008	0.0207	0.0239	0.0313	0.0002
18	0.0059	0.0092	0.0011	0.0177	0.0217	0.0269	0.0004
19	0.006	0.0092	0.0012	0.0177	0.0219	0.0274	0.0001
20	0.0054	0.009	0.0014	0.018	0.0216	0.0268	0.0003
21	0.0061	0.0088	0.0013	0.0179	0.0211	0.0266	0.0003
22	0.0055	0.0085	0.0013	0.0167	0.0203	0.0257	0.0001
23	0.0061	0.0097	0.0013	0.0172	0.021	0.0257	0.0001
24	0.0061	0.0098	0.0009	0.0165	0.02	0.0249	0.0003
25	0.0068	0.0103	0.0011	0.0168	0.0204	0.0258	0.0001
26	0.0065	0.0101	0.001	0.0164	0.0203	0.0254	0.0003
27	0.0071	0.0113	0.0009	0.0179	0.0199	0.0251	0.0002
28	0.0049	0.0094	0.0009	0.0167	0.0175	0.0234	0

FITTING PROCEDURE

To obtain a consistent set of rate parameters, model fitting was conducted for both HDPE and LLDPE simultaneously. Rather than fitting Arrhenius pre-factors, A, and Evans-Polanyi intrinsic barriers, E_0, directly, multiplicative and additive correction factors, θ and λ, were tuned for reaction families that share an analogous transition state structure. For instance, the rate coefficients for all the hydrogen shift reactions are described with:

$$k_{HS} = (\theta \cdot A_{HS}) \cdot e^{\left(\frac{-\left(\left(\lambda + E_{0,HS}\right) + \alpha \cdot \Delta H_{rxn}\right)}{RT}\right)}$$

For the most part, the θ parameters, initialized with a value of one, were tuned within bounds of 0.1 to 10, while the λ parameters, initialized with a value of zero, were tuned within bounds of -3 to 3. However, in order to match the elevated yields of 1-hexene observed experimentally, the tertiary carbon bond fission rates, as well as the abstraction of hydrogen atoms from branch-ends needed to be fit with wider bounds. The optimized values of the correction factor and their corresponding gPROMS confidence intervals are reported in Table S7.

Table S7 Tabulation of the optimized correction factors for various reaction family categories and their corresponding confidence intervals reported by the gPROMS parameter fitting procedure. The hydrogen shift reactions encompass both the end-chain and mid-chain variations of the reaction. For example, the x,x+4-hydrogen shift encompasses the 1,5-hydrogen shift, 5,1-hydrogen shift, and x,x+4-hydrogen shift reactions.

Reaction Family Category	Correction Factor	Optimized Value	Confidence Interval
Hydrogen Abstraction	θ	5.0	93.2
	λ	0.75	48.8
x,x+3-Hydrogen Shift	θ	5.83	1934.8
x,x+4-Hydrogen Shift	θ	8.23	58.2
	λ	1.55	63.3
x,x+5-Hydrogen Shift	θ	4.81	10.3
	λ	0.22	1.6
Mid-Chain Beta-Scission	θ	0.59	69.9
	λ	3.0	146.3

Tertiary Chain Fission	λ	2.5	1915.6
Hydrogen Abstraction (End/Mid-Radical from Allylic End-Chain)	Enthalpy Correction Factor	6.00	2955.5

Comparison of LLDPE Model with experimental Results Continued

Figure S1 and Figure S2 depict the comparison between experimental and simulation results collected after 1, 2, 3, 4, and 5 minutes of pyrolysis time at 673.15 K and 703.15 K, respectively. For each figure, the sub-plots A, B, and C are divided by the product classes: alkanes, alkenes, and dienes. The major product at both temperatures is 1-hexene, produced when a radical, either on a branch-end or chain-end, shifts to the C5 position and subsequently undergoes a beta-scission reaction. The elevated yield of hexane at both temperatures, relative to the other alkane products, is does not have as obvious a mechanistic explanation. If it were only produced via beta-scission of the polymer backbone, it would have a wt% more in line with the other alkane species. However, for it to be the result of branch deconstruction via beta-scission, the branch length would need to be ≥C8, which is not commonly commercially available but is a potential explanation. Finally, for it to be the result of chain fission, there would be insufficient remaining branch content to produce the elevated yields of 1-hexene observed.

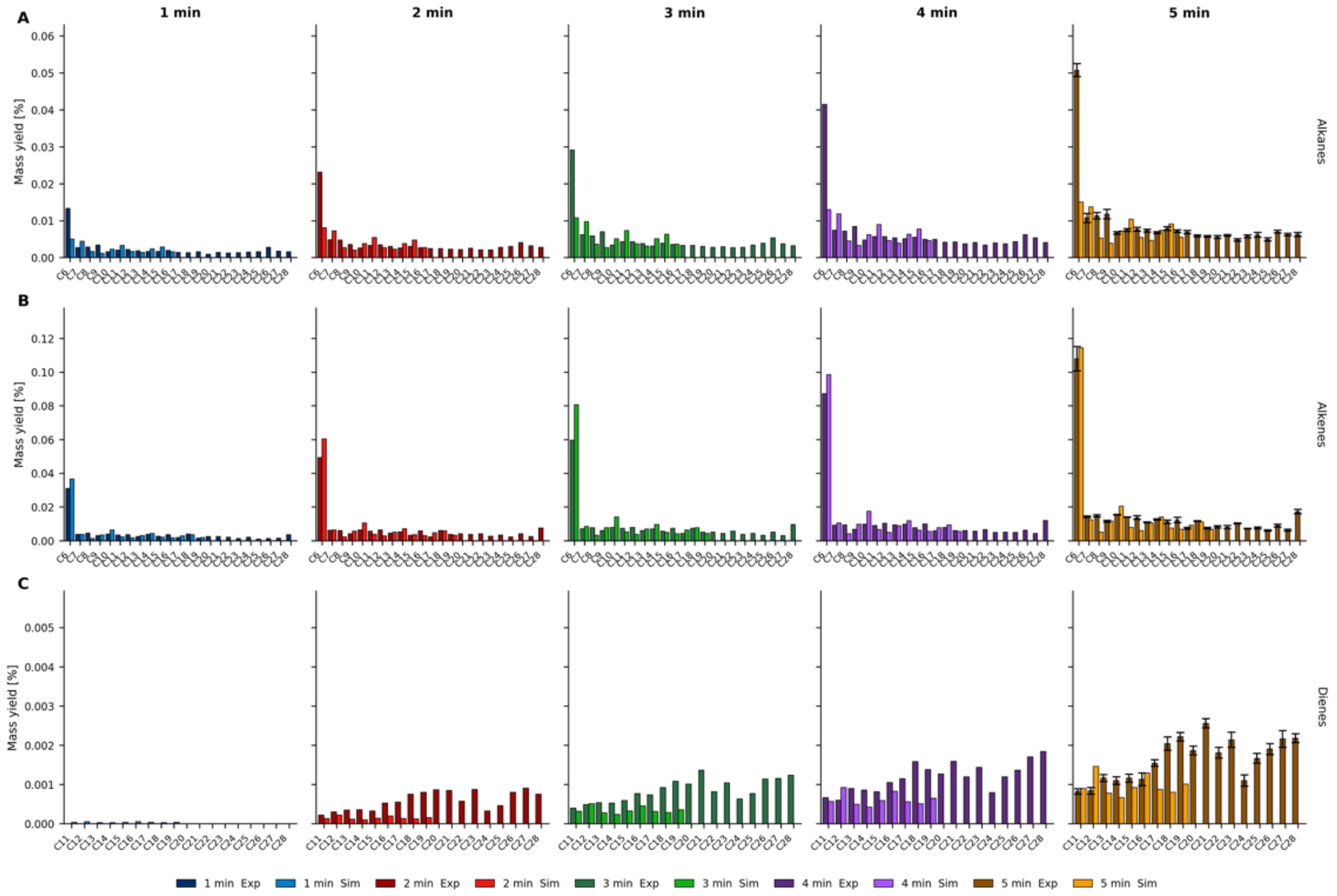

Figure S1 Comparison of simulation results to experimental results after 1, 2, 3, 4, and 5 minutes of pyrolysis time at 673.15 K. Row A depicts alkane mass yields, row B depicts alkene mass yields, and row C depicts diene mass yields.

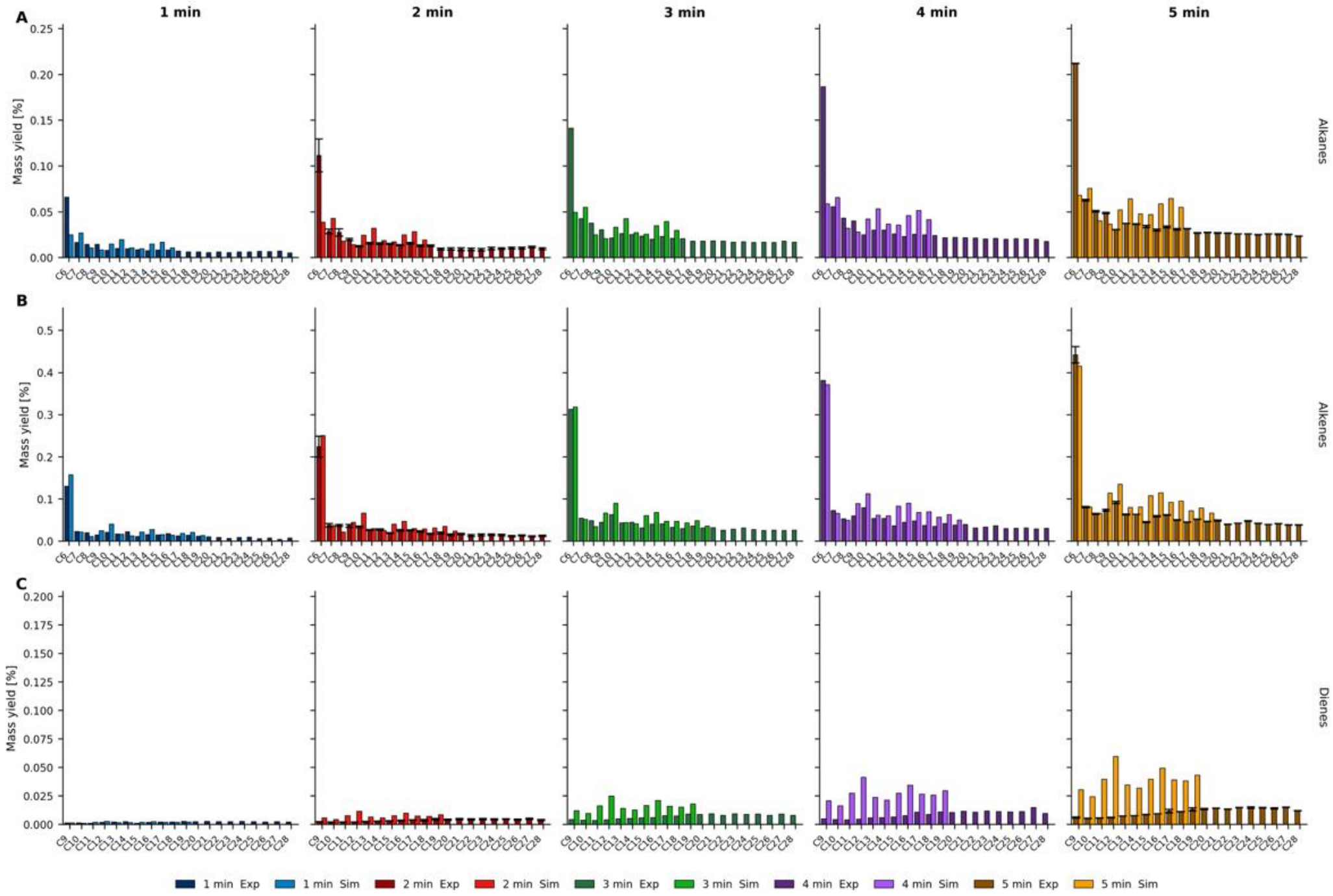

Figure S2 Comparison of simulation results to experimental results after 1, 2, 3, 4, and 5 minutes of pyrolysis time at 703.15 K. Row A depicts alkane mass yields, row B depicts alkene mass yields, and row C depicts diene mass yields.
